# A Traditional Chinese Medicine Plant Extract Prevents Alcohol-Induced Osteopenia

**DOI:** 10.3389/fphar.2021.754088

**Published:** 2021-12-15

**Authors:** Dongyang Qian, Hui Zhou, Pan Fan, Tao Yu, Anish Patel, Morgan O’Brien, Zhe Wang, Shiguang Lu, Guoqiang Tong, Yimin Shan, Lei Wang, Yuan Gao, Yuan Xiong, Lily Zhang, Xin Wang, Yuancai Liu, Shuanhu Zhou

**Affiliations:** ^1^ Department of Orthopedic Surgery, Brigham and Women’s Hospital, Harvard Medical School, Harvard University, Boston, MA, United States; ^2^ Department of Orthopedics, The First Affiliated Hospital, Guangzhou Medical University, Guangzhou, China; ^3^ Jing Brand Research Institute, Jing Brand Co., Ltd., Daye, China; ^4^ Department of Spine Center, Zhongda Hospital, Southeast University Medical School, Nanjing, China; ^5^ Department of Orthopedic Surgery, Tongji Hospital, School of Medicine, Tongji University, Shanghai, China; ^6^ Department of Orthopaedics, Qilu Hospital, Shandong University, Jinan, China; ^7^ Department of Orthopaedics, Union Hospital, Tongji Medical College, Huazhong University of Science and Technology, Wuhan, China; ^8^ Department of Neurosurgery, Brigham and Women’s Hospital, Harvard Medical School, Boston, MA, United States; ^9^ Harvard Stem Cell Institute, Harvard University, Cambridge, MA, United States

**Keywords:** ethnopharmacology, traditional Chinese medicine, alcohol-induced bone diseases, osteoporosis, TCM herbal extracts

## Abstract

Traditional Chinese medicine (TCM) has been practiced in the treatment of bone diseases and alcoholism. Chronic excessive alcohol use results in alcohol-induced bone diseases, including osteopenia and osteoporosis, which increases fracture risk, deficient bone repair, and osteonecrosis. This preclinical study investigated the therapeutic effects of TCM herbal extracts in animal models of chronic excessive alcohol consumption-induced osteopenia. TCM herbal extracts (Jing extracts) were prepared from nine Chinese herbal medicines, a combinative herbal formula for antifatigue and immune regulation, including *Astragalus*, *Cistanche deserticola*, *Dioscorea polystachya*, *Lycium barbarum*, *Epimedium*, *Cinnamomum cassia*, *Syzygium aromaticum*, *Angelica sinensis*, and *Curculigo orchioides*. In this study, Balb/c male mice were orally administrated alcohol (3.2 g/kg/day) with/without TCM herbal extracts (0.125 g/kg, 0.25 g/kg, or 0.5 g/kg) by gavage. Our results showed that after 50 days of oral administration, TCM herbal extracts prevented alcohol-induced osteopenia demonstrated by μ-CT bone morphological analysis in young adults and middle-aged/old Balb/c male mice. Biochemical analysis demonstrated that chronic alcohol consumption inhibits bone formation and has a neutral impact on bone resorption, suggesting that TCM herbal extracts (Jing extracts) mitigate the alcohol-induced abnormal bone metabolism in middle-aged/old male mice. Protocatechuic acid, a natural phenolic acid in Jing extracts, mitigates *in vivo* alcohol-induced decline of alkaline phosphatase (ALP) gene expression in the bone marrow of Balb/c male mice and *in vitro* ALP activity in pre-osteoblast MC3T3-E1 cells. Our study suggests that TCM herbal extracts prevent chronic excessive alcohol consumption-induced osteopenia in male mice, implying that traditional medicinal plants have the therapeutic potential of preventing alcohol-induced bone diseases.

## Introduction

Traditional Chinese medicine has treated various diseases, including bone diseases and alcoholism. Osteoporosis is the most common bone disease, representing a significant public health problem worldwide (Cosman et al., 2014; Wright et al., 2014; Langdahl and Andersen, 2018; Compston et al., 2019). There has been considerable progress in understanding postmenopausal and senile osteoporosis; however, knowledge gaps still exist in understanding osteoporosis/osteopenia caused by alcoholism. The effects of alcohol on human health are complex. Although it is debatable about the benefits of moderate drinking, epidemiological studies suggest that light-moderate alcohol consumption benefits the heart and circulatory system, protects against diabetes, and is associated with lower risks for mortality and cancer in older adults (Howard et al., 2004; Karlamangla et al., 2009; Mostofsky et al., 2016; Kunzmann et al., 2018). However, heavy drinking is detrimental to many organs and tissues and is a significant cause of preventable stroke, heart failure, and death (Garaycoechea et al., 2018; GBD, 2018; Wood et al., 2018; USDA and HHS, 2020; NIAAA., 2021). Alcohol has a complex effect on the adult skeleton depending on age, drinking pattern, and alcohol consumption. Light to moderate alcohol consumption is generally reported to be beneficial or have a neutral impact on bone health in old adults, while chronic excessive drinking induces bone mass loss and osteoporosis, which increases fracture risk, deficient bone repair and causes alcohol-induced osteonecrosis (Turner, 2000; Chakkalakal, 2005; Maurel et al., 2012; Sommer et al., 2013; Mikosch 2014; Gaddini et al., 2016).

TCM-based herbal therapies often use alcohol as a delivery medium for centuries; one class of Chinese herbal medicines is Chinese herbal Liqueur (Xia, 2013; Cheung et al., 2021). The alcoholic drinks of herbal extracts are also used for a wide range of medicinal purposes documented in classical pharmacy and ethnobotanical studies in Europe (Egea et al., 2015). The Chinese medicinal or herbal Liqueur, an alcoholic beverage, is commonly produced by soaking precious Chinese medicinal materials (Xia, 2013; Cheung et al., 2021). It became prevalent among Chinese people due to its nourishing and tonic functions or as nutraceuticals. A typical TCM formula has multiple ingredients that synergize in treatment for the symptoms of a disease, which have greater efficacy and safety than a single drug in clinical practices, possibly due to the multiple ingredients synergistic interactions and mutual detoxification (Ung et al., 2007; He et al., 2015; Zhang and Cheng, 2019). The TCM herbal extracts (Jing extracts) used in this study were prepared from a herbal combination formula of nine Chinese herbal medicines, which was used in a famous Chinese Herbal Liqueur, Chinese Jing Liqueur (Liu et al., 2011; Lu et al., 2017; Lu et al., 2018; Shan et al., 2018; Cai et al., 2020; Hu et al., 2021). Preclinical and clinical studies demonstrated that studied herbal formulation has the properties of improving kidney-yang deficiency in rats and relieving main symptoms of patients with Kidney-Yang Deficiency Syndrome, anti-fatigue, and enhancing immunity in humans and animals (Liu et al., 2011; Lu et al., 2017; Lu et al., 2018; Shan et al., 2018; Cai et al., 2020; Hu et al., 2021). The analyses of chemical components by HPLC, LC/MS, and NMR (Liu et al., 2008; Cai et al., 2020; Hu et al., 2021) showed that the extracts of these Chinese herbal medicines (Jing extracts) contain a variety of saponins, flavonoids, active polysaccharides, and other functional factors and nutrients required by the human body. Although preclinical and clinical data show that saponins, flavonoids, and polysaccharides prevent osteoporosis, the therapeutic effects of Jing extracts on alcohol-induced bone diseases were unknown. In this study, we investigated whether TCM herbal extracts prevent chronic alcohol consumption-induced osteopenia in mice.

## Materials and Methods

### Materials and Experimental Design

**FIGURE 1:**
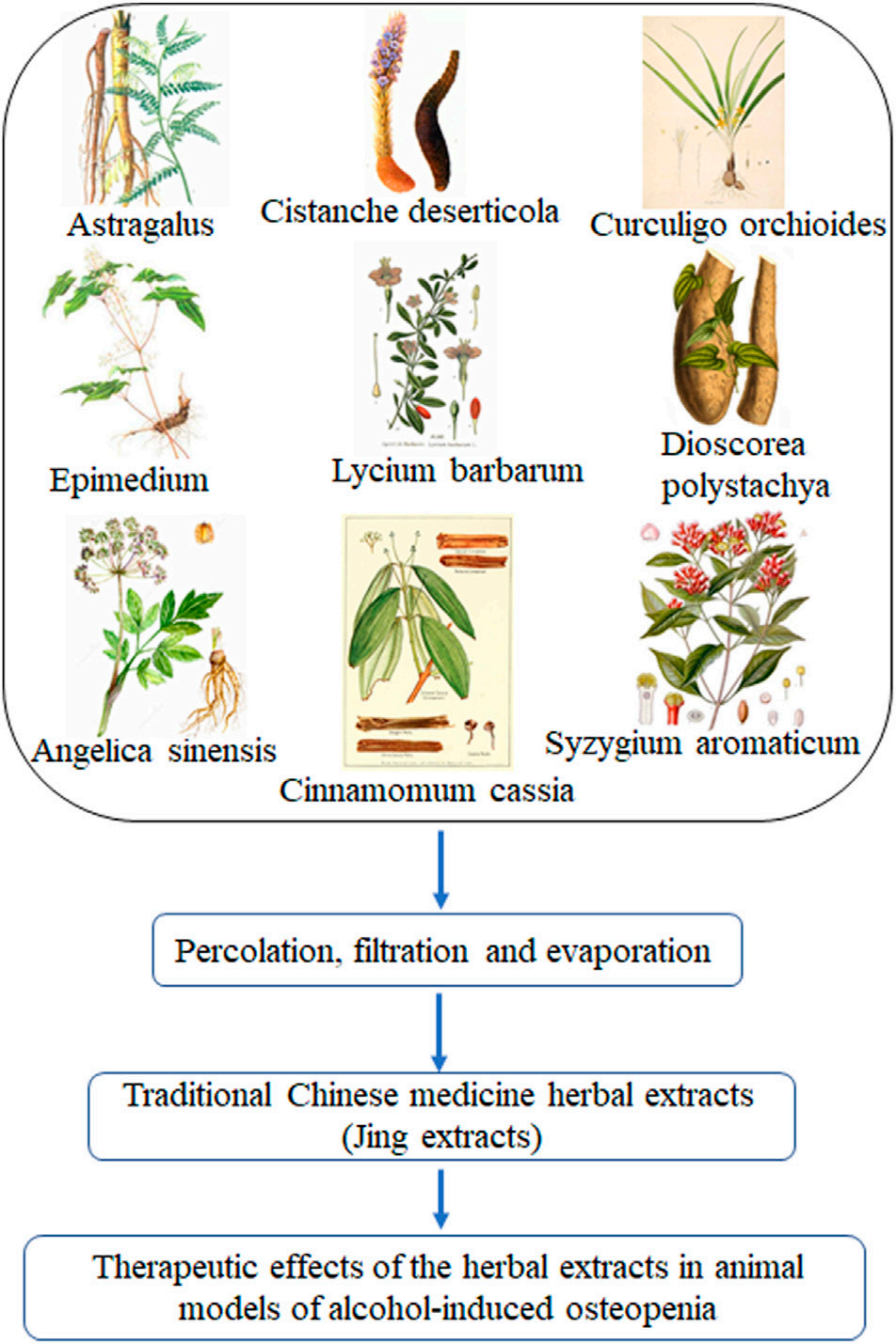
Traditional Chinese medicine herbal extracts (Jing extracts) and experimental design. After percolation, filtration, and evaporation, the concentrated extracts of the nine traditional Chinese medicine herbs, referred to as Jing extracts, were used to discern the therapeutic effects of the herbal extracts in animal models of alcohol-induced osteopenia.

The traditional Chinese medicine herbal extracts used in this study is a combinative herbal formula of nine Chinese herbal medicines, including *Astragalus mongholicus* Bunge (*Astragalus*) (10–15% of total herbal medicine weight as described in Liu et al., 2008), *Cistanche deserticola* Y. C. Ma (*Cistanche deserticola*) (10–15%), *Dioscorea polystachya* Turcz. (*Dioscorea polystachya*, Chinese yam) (10–15%), *Lycium barbarum* L. (*Lycium barbarum*, Chinese wolfberry or Goji berry) (10–15%), *Epimedium brevicornu* Maxim (*Epimedium*, Herba Epimedii) (10–15%), *Cinnamomum cassia* (L.) J. Presl (*Cinnamomum cassia*, Chinese Cinnamon) (3–5%), *Syzygium aromaticum* Merr. and L.M.Perry (*Syzygium aromaticum*) (3–5%), *Angelica sinensis* (Oliv.) Diels (*Angelica sinensis*, Chinese angelica) (10–15%), and *Curculigo orchioides* Gaertn. (*Curculigo orchioides*, *Rhizoma curculiginis*) (10–15%) (Figure 1; Supplementary Figure S1; Table 1; Supplementary Table S1). This herbal formula was originally designed in late China’s Qing Dynasty. After being modified several times by TCM experts in the 1980s, it was approved by China’s National Medical Products Administration (Approval # 1997 No. 728) for anti-fatigue and enhancing immunity as the TCM herbal formula in a famous Chinese Herbal Liqueur, Chinese Jing Liqueur (Liu et al., 2011; Lu et al., 2017; Lu et al., 2018; Shan et al., 2018; Cai et al., 2020; Hu et al., 2021). The traditional Chinese medicine herbs were collected by researchers at Jing Brand Research Institute. Voucher specimens (as described in Cai et al., 2020) are deposited at the Herbarium of Jing Brand Research Institute, Daye, Hubei, China. The raw Chinese herbal medicines were washed, dried, and sliced into pieces and superfine pulverization according to the protocols in Chinese Pharmacopoeia. The traditional Chinese medicine herbal extracts (Jing extracts) were prepared with a percolation extraction method with 35% alcohol (50-fold of total herbal medicine weight for 12–15 days) (Liu et al., 2008; Shan et al., 2018; Cai et al., 2020). After percolation, the liquids were separated from insoluble materials with disc centrifuge and membrane separation technology and concentrated via nanofiltration membrane; the concentrated traditional Chinese medicine herbal extracts, referred to as Jing extracts, was obtained for pharmacognostic analysis to discern the effects of Jing extracts on alcohol-induced osteopenia (Figure 1). The extracts of these Chinese herbal medicines (Jing extracts) have been characterized by chemical constituent analysis with LC/MS and NMR (Cai et al., 2020; Hu et al., 2021) as well as high-performance liquid chromatogram-gas chromatography fingerprint analysis (Liu et al., 2008) (Table 2; Supplementary Figure S7).

**TABLE 1:** The roles of the studied herbs in bone diseases and alcohol use disorder.

Herbs^a^	Roles of herbs in bone diseases	Roles of herbs in alcohol use disorder
*Astragalus mongholicus* Bunge (*Astragalus*) Chinese: Huang Qi	*Astragalus* with supplemental calcium significantly improved bone mineral density and bone metabolism in ovariectomized rats Kang et al. (2013)	The extracts from *Myristica fragrans*, *Astragalus*, and Poria cocos ameliorated alcohol-induced acute liver toxicity Yimam et al. (2016)
*Cistanche deserticola* Y. C. Ma (Cistanche deserticola) Chinese: Rou Cong Rong	*Cistanche deserticola* has antiosteoporotic activity in type I osteoporotic rats Zhang et al. (2021) and type II osteoporotic mice Wang et al. (2021)	*Cistanche deserticola* possessed hepatoprotective activity against chronic hepatic injury induced by alcohol Guo et al. (2016)
*Dioscorea polystachya* Turcz. (*Dioscorea polystachya*, Chinese yam) Chinese: Shan Yao	*Dioscorea spongiosa* prevents glucocorticoid-induced osteoporosis in rats Han et al. (2016) and aging-induced osteoporosis Tikhonova et al. (2015)	*Dioscorea polystachya* extracts have protective effects against alcohol-induced gastric ulcers in mice Byeon et al. (2018)
*Lycium barbarum* L. (*Lycium barbarum*, Chinese wolfberry or Goji berry) Chinese: Gou Qi	*Lycium barbarum* ameliorates osteoporosis in OVX mice Kim et al. (2017)	*Lycium barbarum* has a hepatoprotective effect against alcohol-induced oxidative damage Wang et al. (2020a)
*Epimedium brevicornu* Maxim (Epimedium, Herba Epimedii) Chinese: Yin Yang Huo	*Epimedium* reverses bone loss in a rat model of postmenopausal osteoporosis Zhang et al. (2006) and alcohol-induced osteopenia Wu et al. (2018)	Icariin, one of the bioactive compounds in epimedium, may reduce alcohol consumption in bipolar disorder and alcohol use Xiao et al. (2016)
*Cinnamomum cassia* (L.) J. Presl (*Cinnamomum cassia*, Chinese Cinnamon), Chinese: Rou Gui	*Astragalus membranaceus*, *Cinnamomum cassia*, and *Phellodendron amurense* reverse bone loss in postmenopausal osteoporosis Huh et al. (2015)	Cinnamon extract protects against acute alcohol-induced liver steatosis in mice Kanuri et al. (2009)
*Syzygium aromaticum* Merr. and L.M.Perry (Syzygium aromaticum, clove) Chinese: Ding Xiang	*Syzygium aromaticum* reverses bone loss in a rat model of postmenopausal osteoporosis Karmakar et al. (2012)	*Syzygium aromaticum* attenuates alcohol-induced gastric injury in rats Jin et al. (2016)
*Angelica sinensis* (Oliv.) Diels (*Angelica sinensis*, Chinese angelica) Chinese: Dang Gui	*Angelica sinensis* reverses bone loss in a rat model of postmenopausal osteoporosis Lim and Kim, (2014)	*Angelica sinensis* protects against alcoholic liver damage in mice Wang et al. (2020a)
*Curculigo orchioides* Gaertn. (*Curculigo orchioides*, *Rhizoma curculiginis*, Curculigo Rhizome) Chinese Pinyin: Xian Mao	*Curculigo orchioides* prevents bone loss in ovariectomized rats Cao et al. (2008)	*Curculigo orchioides* extract has antioxidant and hepatoprotective properties in rats Jacob Jesurun and Johan Pandian, (2019)

a The Chinese medicinal plant names referred to The Plant List (http://www.theplantlist.org/), Medicinal Plant Names Services of Royal Botanic Gardens, Kew (https://mpns.science.kew.org/mpns-portal/), or NCBI taxonomy database (https://www.ncbi.nlm.nih.gov/taxonomy).

**TABLE 2:** Roles of bioactive compounds in the studied herbs in bone diseases and alcohol use disorder.

Bioactive compounds^a^ ^,^ ^b^	The herbs contain the compound^c^	Roles of bioactive compounds in bone diseases	Roles of bioactive compounds in alcohol use disorder
Acteoside	*Cistanche deserticola*	Acteoside has a protective effect on OVX-induced bone loss in mice Yang et al. (2019a)	Acteoside ameliorates alcohol-induced hepatic damage Khullar et al. (2019)
Calycosin	*Astragalus Angelica sinensis*	Calycosin has an unusual antiosteoporotic activity in OVX rats Li et al. (2016)	
Daidzin	*Astragalus*	Daidzin is a promising agent in the treatment of osteolytic diseases Wei et al. (2019)	Daidzin is an antidipsotropic agent Keung and Vallee, (1998)
Echinacoside	*Cistanche deserticola*	Echinacoside is especially effective for preventing osteoporosis induced by estrogen deficiency Li et al. (2013)	Echinacoside ameliorates alcohol-induced oxidative stress and hepatic steatosis Tao et al. (2021)
Epimedin A	*Epimedium*	Epimedin A remarkably enhances bone regeneration Liu et al. (2021)	
Eugenol	*Cistanche deserticola Syzygium aromaticum*	*Syzygium aromaticum* extract eugenol reverses bone loss in a rat model of postmenopausal osteoporosis Karmakar et al. (2012)	Eugenol reduced alcohol-induced hepatotoxicity in rats Yildiz and Öztürk, (2020)
Ferulic acid	*Angelica sinensis Lycium barbarum*	Ferulic acid protects against osteoporosis in neonatal rats with glucocorticoid-induced osteoporosis Hou et al. (2019)	Ferulic acid is an anti-hepatotoxic agent on alcohol-induced liver damage Rukkumani et al. (2004)
Hyperoside	*Epimedium*	Hyperoside has potential beneficial effects on bone metabolism in OVX mice Chen et al. (2018)	Hyperoside is one of the polyphenols extracted from a white tea that has preventative effects against alcoholic liver injury Zhou et al. (2019)
Icariside	*Epimedium*	Icariside II might treat dexamethasone-associated osteoporosis/osteonecrosis Liu et al. (2017)	
Icariin	*Epimedium*	Icariin has the potential treatment of postmenopausal osteoporosis Wang et al. (2018)	Icariin may resduce alcohol consumption in people with bipolar disorder and alcholol use Xiao et al. (2016)
Oleanolic acid	*Syzygium aromaticum*	Oleanolic acid significantly increased bone mineral density in OVX mice Cao et al. (2018)	Oleanolic acid protect rats against ethanol-induced liver injury Liu et al. (2014)
Protocatechuic acid	*Angelica sinensis*	Protocatechuic Acid attenuates bone loss in OVX mice Jang et al. (2018)	Protocatechuic acid alleviates alcholic liver injury Fu et al. (2019)
Sagittatoside A and B	*Epimedium*	Sagittatoside A and B might be beneficial for improving postmenopausal osteoporosis Zhao et al. (2016)	
(Z)-Ligustilide	*Angelica sinensis*	Ligustilide promotes bone formation Yang et al. (2019b)	

a Among the 189 compounds by LC/MS and NMR (Cai et al., 2020) and 43 bioactive components by LC/MS (Hu et al., 2021) isolated from the Chinese herbal extracts.

b The chemical structures of herbal bioactive compounds in Supplementary Figure S7.

c Reference to Zeng et al. (2019) and Fai (2009).

### Animal Models of Alcohol-Induced Osteopenia

Two-month-old Balb/c male mice were purchased from Jackson lab (Bar Harbor, ME) as the young adult animal in this study (after 50 experimental periods, the mice were 4-month-old). The middle-aged/old Balb/c male mice (10-month-old) (after 50 experimental periods, the mice were about 12-month-old) were purchased from Envigo (Indianapolis, IN). Mice were provided with standard chow and water *ad libitum*. Animal maintenance and experimental treatments were conducted per the ethical guidelines for animal research established and approved by BWH Institutional Animal Care and Use Committee.

**FIGURE 2:**
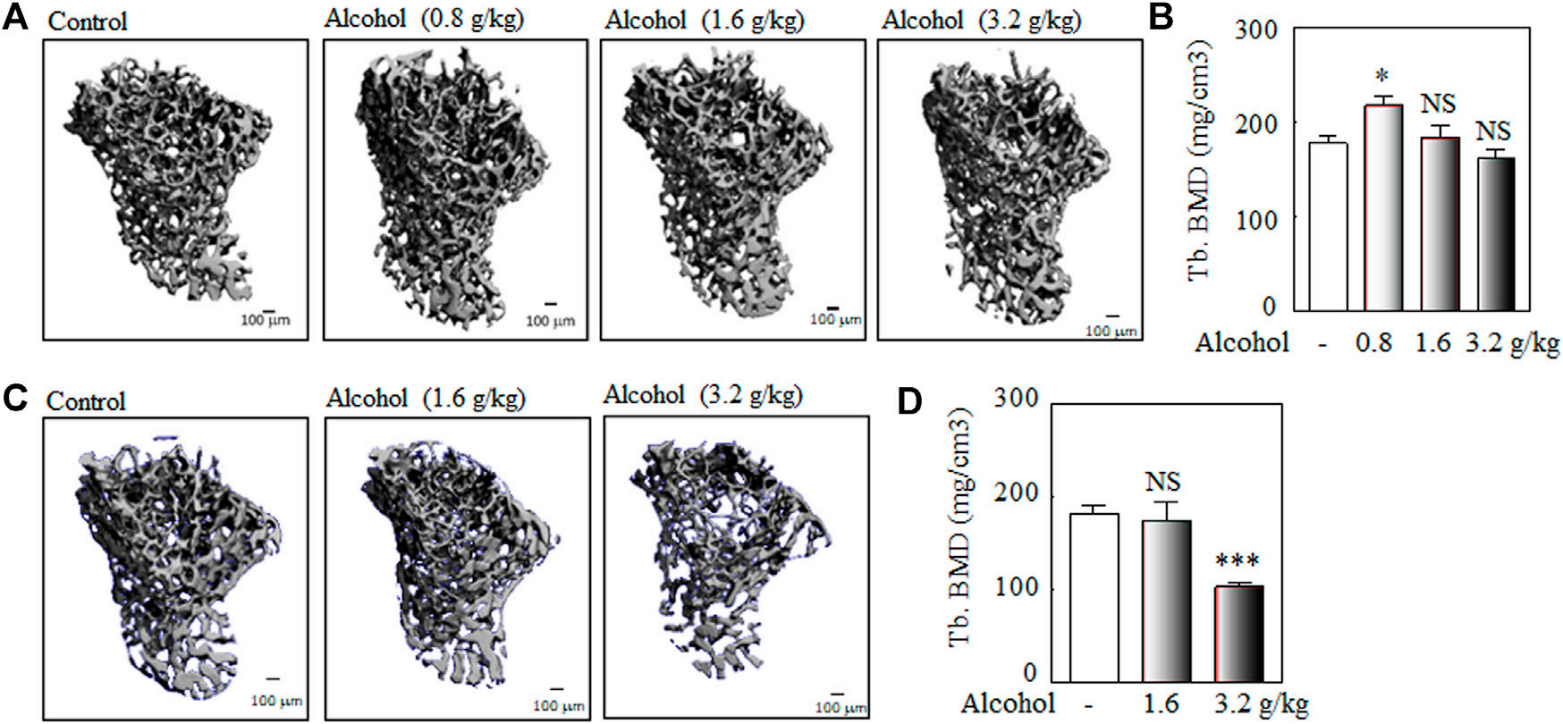
The murine models of alcohol-induced osteopenia: the dose and duration. Balb/c male mice (2-month-old) were used to explore the optimal dose and duration of the oral alcohol administration by gavage for alcohol-induced osteopenia. The young adult Balb/c male mice (2-month-old) were used to analyze the optimal dose (0.8, 1.6 g/kg, and 3.2 g/kg body weight) and duration (once per day at 3–4 PM, 5 days per week for 30 or 50 days) of alcohol-induced osteopenia. **(A)** The representative μ-CT 3-D microstructures of trabecular bone were obtained from male mice with alcohol gavage (Alcohol) or water gavage (Control) for 30 days; bars represent 100 μm. 3-D microstructural properties of the tibia were calculated using software supplied by the manufacturer. **(B)** The quantitative analysis of trabecular bone mineral density (Tb. BMD); the oral alcohol administration of 0.8 g/kg dose of alcohol (*n* = 4) for 30 days significantly increased BMD in Balb/c adult male mice (alcohol vs. control group, *n* = 5, *p < 0.05, Mann-Whitney test); the 1.6 g/kg (*n* = 7) and 3.2 g/kg (*n* = 5) doses of alcohol for 30 days did not induce significant bone loss (alcohol vs. control group, *n* = 5; NS: not significant, Mann-Whitney test). **(C)** The representative μ-CT 3-D microstructures of trabecular bone were obtained from male mice with alcohol gavage (Alcohol) or water gavage (Control) for 50 days. **(D)** The quantitative analysis of trabecular bone mineral density (Tb. BMD); a lower dose (1.6 g/kg) of alcohol for a longer duration (50 days) did not induce bone loss (alcohol, *n* = 7, vs. control, *n* = 18, NS: not significant, Mann-Whitney test); the oral alcohol administration of 3.2 g/kg alcohol for 50 days induced significant bone loss (alcohol, *n* = 8 vs. control group, *n* = 18, ***p < 0.001, *t*-test).

There are several different murine models of alcohol-induced bone loss regarding alcohol administration, including oral alcohol administration by gavage (Iwaniec and Turner, 2013; Martiniakova et al., 2018), alcohol in drinking water (Liu et al., 2016; Okuda et al., 2018), intraperitoneal (i.p.) administration of alcohol by injection (Iwaniec and Turner, 2013), and Liber-DeCarli liquid diet feeding (Dai et al., 2000; Mercer et al., 2012). Although there are advantages and disadvantages among the methods of alcohol administration, oral alcohol administration by gavage in animals is a better model to mimic intermittent drinking in humans. We used the oral alcohol administration by gavage in Balb/c male mice as the murine models of alcohol-induced osteopenia and to study the effects of TCM extracts on alcohol-induced osteopenia. The process of oral alcohol administration by gavage was performed as described in the NIAAA model (Bertola et al., 2013). As shown in Figure 2, oral administration of 3.2 g/kg alcohol (0.2 ml of 40% v/v alcohol for a 20 g body weight mouse) by gavage for 50 days was used to study alcohol-induced osteopenia in male Balb/c mice.

### Traditional Chinese Medicine Herbal Extracts (Jing Extracts) Administration in Mice

The doses of Jing extracts used in the experimental mice were designed based on the recommended human dose (Liu et al., 2008; Liu et al., 2011; Lu et al., 2017; Lu et al., 2018; Shan et al., 2018) and the human-mouse dose conversion (Wojcikowski and Gobe, 2014; Nair and Jacob S., 2016) (details refer to Supplementary Material). Taking into account that the commonly used mouse gavage volume is 10 ml/kg (0.2 ml for a 20 g mouse), we defined the first mouse dose of Chinese herbal extracts (0.2 ml for a 20 g mouse), as the low dose (0.125 g/kg body weight for a 20 g mouse with 0.2 ml oral administration by gavage of 12.5 g/L of Chinese herbal extracts in 40% v/v alcohol); the second and third doses contained 0.25 g/kg (25 g/L of Chinese herbal extracts in 40% v/v alcohol) and 0.50 g/kg (50 g/L of Chinese herbal extracts in 40% v/v alcohol) of Chinese herbal extracts respectively. We tested the effects of these three doses of Chinese herbal extracts on alcohol-induced bone loss in Balb/c male mice. After 50 days of orally administering 3.2 g/kg of alcohol with/without Chinese herbal extracts by gavage, the mice were sacrificed for bone morphological and biochemical analysis.

### Bone Morphological Analysis: μ-CT

Bone morphological analysis of proximal tibia was performed with a μ-CT (μ-CT 35, Scanco Medical, Switzerland) as described (Charles et al., 2012). The scanned region proximal to the growth plate and extending 1.4 mm was selected for trabecular bone analysis (indicated with a box Supplementary Figure S2). A second region 0.6 mm in length and centered at the midpoint of the tibia was used to calculate the diaphyseal parameters (shown with a box in Supplementary Figure S2). 3-D microstructural properties of bone, e.g., trabecular bone mineral density (Tb. BMD), tibia trabecular relative bone volume (Tb. BV/TV), trabecular thickness (Tb. Th), trabecular space (Tb. Sp), trabecular number (Tb. N), and cortical bone mineral density (C. BMD), were calculated using software supplied by the manufacturer (Scanco Medical, Switzerland).

### Bone Morphological Analysis: Histological Analysis

In addition to μ-CT analysis of skeletal parameters, some murine bones were used for histological analysis. The tibia was removed from the attached muscle tissue, fixed with 10% formalin (at least 48 h), decalcified with EDTA, plastic-embedded (JB-4 Kit, Polysciences, Warrington, PA), and sectioned (10 μm); the sections were stained with 0.5% toluidine blue-O, pH 4.0 (Thermo Fisher Scientific, Waltham, MA, United States).

### Biochemical Analysis of Alcohol-Induced Bone Loss

The blood was taken 6 h after fasting on the second day of the last gavage, left for 30–60 min after blood collection, centrifuged at 1,500 g for 15 min, and the serum was separated and kept at −80 C until the biochemical analysis for biomarkers of bone formation, e.g., osteocalcin (OC), and bone resorption, e.g., Type I collagen carboxy-terminal telopeptide (CTX). The serum levels of osteocalcin, a non-collagenous protein formed by osteoblasts, were detected by using the mouse osteocalcin ELISA kit (MyBioSource, San Diego, CA, United States). Type I collagen is the main organic component of bones. When physiological or pathological bone resorption is increased (such as osteoporosis), the degradation of type I collagen is also increased, and the content of decomposed fragments in the blood is correspondingly increased. The main molecular fragment of type I collagen degradation products is the CTX. An increase in the serum CTX level indicates an increase in bone resorption. The serum levels of Type I collagen carboxy-terminal telopeptide (CTX) were detected by using Mouse Cross-Linked C-Telopeptide of Type I Collagen (CTXI) ELISA Kit (MyBioSource, San Diego, CA, United States).

### RNA Isolation and RT-PCR

Total RNA was isolated from bone marrow or MC3T3-E1 cells with Trizol reagent (Invitrogen). For RT-PCR, 2 μg of total RNA was reverse-transcribed into cDNA with M-MLV reverse transcriptase (Promega, WI), following the manufacturer’s instructions. One-twentieth of the cDNA was used in each 50 μl PCR reaction with Promega GoTaq Flexi DNA Polymerase (30–35 cycles of 94°C for 1 min, 55–60°C for 1 min, and 72°C for 2 min). Amplification conditions were optimized to reflect the exponential phase of amplification for each gene. Gene-specific primers were used to amplify ALP, TNF-α, IL-1β, BSP, RUNX2, and BMP2 as shown in Supplementary Table S2. PCR products were separated by 2% agarose gel electrophoresis and captured with KODAK Gel Logic 200 Imaging System and KODAK Molecular Imaging Software (Carestream Health, Rochester, NY, United States). The densitometry of inverted ethidium bromide staining PCR bands was quantified by NIH ImageJ. Relative expression levels were calculated by normalizing the densitometric units to GAPDH, the internal control.

### Alkaline Phosphatase Enzyme Assay

MC3T3-E1 cells were cultured in quadruplicate in 24-well-plates in a growth medium. Upon confluence, medium was changed to osteoblastogenic medium (phenol red-free α-MEM with 10% FBS-HI, 100 U/ml penicillin, 100 μg/ml streptomycin plus 5 mM β-glycerophosphate and 50 μg/ml ascorbate-2-phosphate) for 6 days. Protein concentrations were quantified using BCA Assay kit, and ALP enzyme activity was measured spectrophotometrically and expressed as μmol/min/g protein (Zhou et al., 2008).

### Statistical Analysis

All experiments were performed at a minimum in triplicate; for *in vivo* experiments, a minimum three mice per experimental group per time were performed; for *in vitro* ALP activity, quadruplicate wells per experimental group were performed. All test parameters were quantified as continuous data and used standard statistical tests. The Kolmogorov-Smirnov tool was used to test whether data sets were distributed normally. Group data are presented as mean ± SEM. Unless otherwise indicated, quantitative data were analyzed with a non-parametric Mann-Whitney test or if data allowed, a parametric *t*-test for group comparisons with GraphPad Instat (GraphPad Software, La Jolla, CA, United States). A value of *p* < 0.05 was considered significant.

## Results

### The Murine Model of Alcohol-Induced Osteopenia: The Optimal Dose and Duration of Oral Alcohol Administration

The young adult Balb/c male mice (2-month-old) were used to explore the doses (0.8, 1.6, and 3.2 g/kg body weight, once per day at 3–4 PM, 5 days per week) and duration (30 and 50 days) of oral alcohol administration for alcohol-induced osteopenia in male mice. Our data showed that Balb/c male mice (2-month-old) were orally administered alcohol by gavage, the 0.8 g/kg dose of alcohol for 30 days significantly increased BMD in Balb/c adult male mice, and the 1.6 g/kg and 3.2 g/kg alcohol did not induce significant bone loss after 30 days gavage (Figures 2A,B); a low dose of 1.6 g/kg alcohol did not affect bone mineral density after 50 days gavage (Figures 2C,D); after 50 days of oral administered alcohol, 3.2 g/kg of alcohol-induced significant bone loss in Balb/c male mice (about 4-month-old when sacrificed) (Figures 2C,D). Thereafter, we used the dose of 3.2 g/kg alcohol and a duration of 50 days for the effects of TCM extracts on alcohol-induced osteopenia.

### Chinese Herbal Extracts Prevent Chronic Alcohol Consumption-Induced Osteopenia in Young Adult Male Mice

**FIGURE 3:**
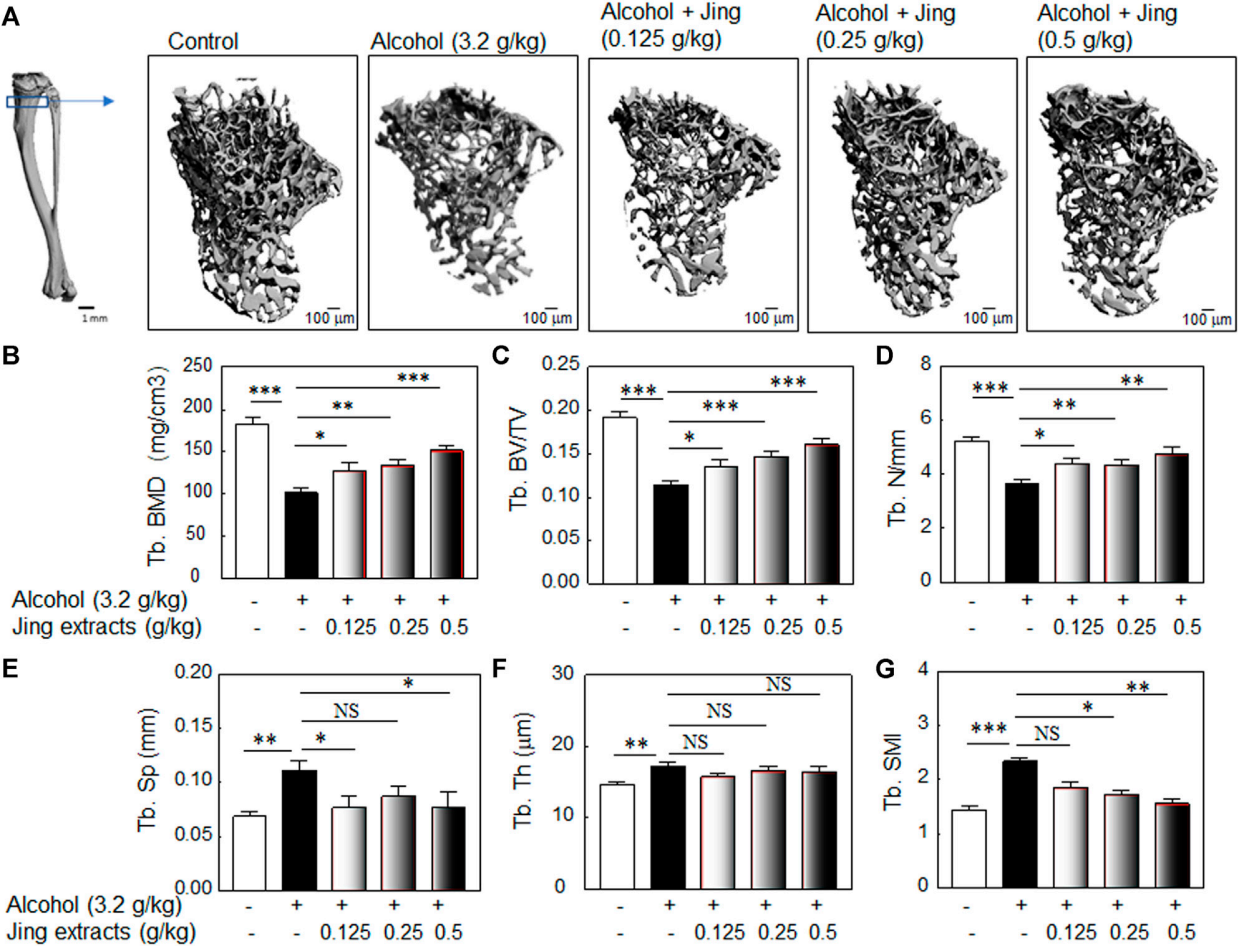
Traditional Chinese medicine herbal extracts (Jing extracts) prevent chronic alcohol consumption-induced osteopenia in young adult male mice. To explore the optimal dose of Jing extracts that prevent alcohol-induced osteopenia in young adult mice, Balb/c male mice (2-month-old) were orally administered 10 ml/kg of 40% alcohol (3.2 g/kg body weight) by gavage for 50 days (gavage once a day, 5 days per week) with or without 0.125 g/kg, 0.25 g/kg or 0.5 g/kg of Jing extracts. **(A)** The representative μ-CT 3-D microstructures of trabecular bone were obtained from male mice with alcohol gavage (Alcohol) ± Jing extracts or without alcohol (Control); bars represent 100 μm. 3-D microstructural properties of the tibia were calculated using software supplied by the manufacturer. **(B)** The quantitative analysis of trabecular bone mineral density (Tb. BMD) (3.2 g/kg of Alcohol, *n* = 9, vs. DDW control, *n* = 19, ***p < 0.001, *t*-test; Alcohol + 0.125 g/kg of Jing extracts, *n* = 4, vs. Alcohol, *n* = 9, *p < 0.05, Mann-Whitney test; Alcohol + 0.25 g/kg of Jing extracts, *n* = 11 vs. Alcohol, *n* = 9, **p < 0.01, *t*-test; Alcohol + 0.5 g/kg of Jing extracts, *n* = 5, vs. Alcohol, *n* = 9, ***p < 0.001, *t*-test). **(C)** The quantitative analysis of tibia trabecular relative bone volume (Tb. BV/TV) (***p < 0.001, *t*-test; *p < 0.05, Mann-Whitney test). **(D)** The quantitative analysis of tibia trabecular number per mm (Tb. N) (***p < 0.001, **p < 0.01, *t*-test; *p < 0.05, Mann-Whitney test). **(E)** The quantitative analysis of tibia trabecular separation (mm) (**p < 0.01, *t*-test; *p < 0.05, NS: not significant, Mann-Whitney test). **(F)** The quantitative analysis of trabecular thickness (Tb. Th, μm) (**p < 0.01, *t*-test; NS: not significant, Mann-Whitney test). **(G)** The quantitative analysis of tibia trabecular Structure modulus index (Tb. SMI) (***p < 0.001, **p < 0.01, *t*-test; NS: not significant, Mann-Whitney test).

We used the dose of 3.2 g/kg of alcohol administered by gavage for 50 days, which induced significant bone loss in young adult male Balb/c mice, as shown in Figure 2, for the effects of herbal extracts on alcohol-induced osteopenia. To explore the optimal doses of traditional Chinese medicine herbal extracts (Jing extracts) that prevent alcohol-induced osteopenia, Balb/c male mice (2-month-old) were orally administered 3.2 g/kg alcohol by gavage with or without 0.125 g/kg, 0.25 g/kg, or 0.5 g/kg of traditional Chinese medicine herbal extracts (Jing extracts) (as described in Figure 1). After 50 days of oral administration of alcohol with/without Chinese herbal extracts by gavage, the mice were sacrificed for bone morphological analysis by μ-CT with the methods described in Supplementary Figure S2 and Figure 3. The 3-D microstructures of trabecular bone (Figure 3A) and quantitative analysis of the microstructural properties of the tibia (Figures 3B–G), including trabecular bone mineral density (Tb. BMD), trabecular relative bone volume (Tb. BV/TV), trabecular number per mm (Tb. N), and trabecular Structure modulus index (Tb. SMI), showed that the effect of Chinese herbal extracts is dose-dependent in preventing alcohol-induced bone loss in young adult male mice. Our data showed that 3.2 g/kg alcohol induced significant bone loss in young adult male Balb/c mice after 50 days of oral administration by gavage (Figure 3); the low, middle, and high doses of Chinese herbal extracts alleviate the chronic alcohol consumption-induced trabecular bone damage; all three doses of Chinese herbal extracts mitigate the alcohol-induced decreases of bone mineral density and relative bone volume or bone volume fraction (Figures 3B,C); the Chinese herbal extracts also alleviate alcohol-reduced trabecular number decrease (Figure 3D) and the corresponding increase of trabecular separation (Figure 3E), but no effects were observed for trabecular thickness (Figure 3F). Although there are controversial in the literature (Salmon et al., 2015), structure model index (SMI), which is widely used to measure rods and plates in trabecular bone, represents the trabecular geometry transitions from being more plate-like (reflected high mechanical stress) to more rod-like (low mechanical stress) as osteoporosis severity increases (Ding et at., 2002; Hildebrand and Rüegsegger, 1997), which inversely correlated with the bone mechanical property (Ding et al., 2002). Our data showed that two doses of 0.25 g/kg and 0.5 g/kg of Chinese herbal medicines significantly mitigate the alcohol-induced damage in the bone mechanical property (Figure 3G).

### Traditional Chinese Medicine Herbal Extracts (Jing Extracts) Prevent Chronic Alcohol Consumption-Induced Osteopenia in Middle-Aged/Old Male Mice

Prevention of problematic alcohol use is mainly focused on younger adults, while an epidemiological study showed that heavy drinking in middle-aged and older adults is more frequent with a greater impact on function and health (Veerbeek et al., 2019). There are age-related changes in bone structure and strength in Balb/c mice (Willinghamm et al., 2010). To investigate the preventing effects of Jing extracts on chronic alcohol consumption-induced osteopenia, we used 10-month-old Balb/c male mice (about 12 months old after the experiments of chronic alcohol consumption-induced osteopenia), which have a significantly lower bone mineral density than the young adult mice (about 4 months old after the experiments) (Supplementary Figure S3), but still have enough trabecular bones, as shown by μ-CT and histological staining, to study the chronic alcohol consumption-induced bone loss and the preventive effects of herbal extracts on osteopenia.

**FIGURE 4:**
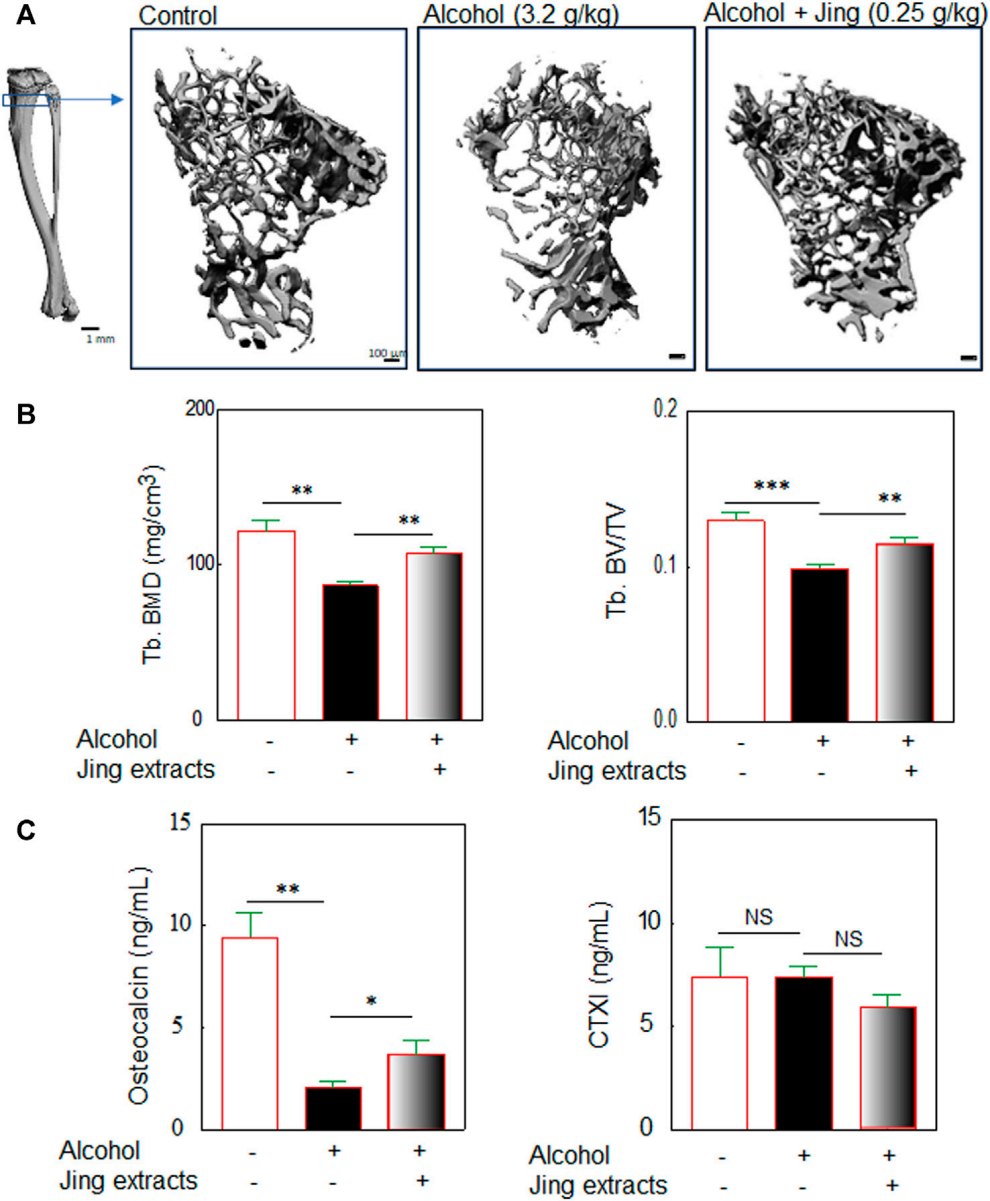
Traditional Chinese medicine herbal extracts (Jing extracts) prevent chronic alcohol consumption-induced osteopenia in middle-aged/old male mice. Balb/c male mice (10-month-old) were orally administered 3.2 g/kg body weight of alcohol by gavage for 50 days (gavage once a day, 5 days per week) with or without 0.25 g/kg of Jing extracts. **(A)** The representative μ-CT 3-D microstructures of trabecular bone were obtained from male mice with alcohol gavage (Alcohol) ± Jing extracts or without alcohol (Control); bars represent 100 μm. 3-D microstructural properties of the tibia were calculated using software supplied by the manufacturer. **(B)** The quantitative analysis of trabecular bone mineral density (Tb. BMD) (3.2 g/kg of Alcohol, *n* = 8, vs. DDW control, *n* = 9, **p < 0.01; Alcohol + 0.25 g/kg of Jing extracts, *n* = 9, vs. Alcohol, *n* = 8, **p < 0.01, *t*-test) and tibia trabecular relative bone volume (Tb. BV/TV) (***p < 0.001, **p < 0.01, *t*-test). **(C)** The effects of Jing extracts on biomarkers of bone formation and bone resorption in middle-aged/old Balb/c male mice. Balb/c male mice (10-month-old) were orally administered 3.2 g/kg body weight of alcohol by gavage for 50 days (gavage once a day, 5 days per week) with or without 0.25 g/kg of Jing extracts (about 12-month-old when mice were sacrificed). Biochemical analysis of bone formation biomarker osteocalcin in serum of middle-aged/old mice (alcohol, *n* = 6, vs. control, *n* = 9, **p < 0.01, *t*-test; Jing extracts, *n* = 7, vs. alcohol, *n* = 6, *p < 0.05, Mann-Whitney test) and biochemical analysis of bone resorption biomarker cross-linked C-Telopeptide of Type I Collagen (CTXI) (NS: not significant, *t*-test).

To study the effects of traditional Chinese medicine herbal extracts on chronic alcohol consumption-induced osteopenia in middle-aged mice, Balb/c male mice (10-month-old) were orally administered 3.2 g/kg alcohol by gavage with or without 0.25 g/kg of Chinese herbal extracts (as described in Figure 1). After 50 days of oral administration of alcohol by gavage with/without Chinese herbal extracts (the mice were about 12-month-old at the end of the experiments), the mice were sacrificed for bone morphological analysis with μ-CT as the methods described in Supplementary Figure S2, including the 3-D microstructures of trabecular bone (Figure 4A) and quantitative analysis of the microstructural properties of the tibia, including trabecular bone mineral density (Tb. BMD) (Figure 4B) and trabecular relative bone volume (Tb. BV/TV) (Figure 4B). Our data showed that alcohol (3.2 g/kg) induced significant bone loss in middle-aged/old male Balb/c mice after 50 days of oral administration by gavage (Figure 4); the 0.25 g/kg of Chinese herbal extracts alleviate the chronic alcohol consumption-induced trabecular bone damage, which was demonstrated by Chinese herbal extracts mitigating the alcohol-induced decreases of bone mineral density and relative bone volume or bone volume fraction (Figure 4B).

The biochemical analysis for biomarkers of bone formation and bone resorption was performed by using the serum obtained from the middle-aged/old mice. The serum levels of osteocalcin, a bone formation biomarker, were detected with a mouse osteocalcin ELISA kit (MyBioSource, San Diego, CA, United States). An increase in the serum type I collagen carboxy-terminal telopeptide (CTX) level indicates increased bone resorption. The serum levels of Type I collagen carboxy-terminal telopeptide (CTX) were detected by using a Mouse Cross-Linked C-Telopeptide Of Type I Collagen (CTXI) ELISA Kit (MyBioSource, San Diego, CA, United States). Our biochemical analysis (Figure 4C) showed that 3.2 g/kg of alcohol significantly reduced serum levels of osteocalcin (Figure 4C) but did not reduce serum levels of CTXI (Figure 4C) in middle-aged/old Balb/c male mice. Out data suggest that chronic alcohol consumption inhibits bone formation but has no effects on bone resorption, which is consistent with previous reports (summarized in Sampson, 1998), indicated that alcoholic osteoporosis is characterized by decreased bone formation but normal levels of resorption. The Chinese herbal extracts mitigate the alcohol-induced decreases of osteocalcin, a bone formation biomarker (Figure 4C), implying that phytomedicines may prevent alcohol-induced osteopenia *via* reducing the alcohol-induced damage in osteoblastogenesis.

### Protocatechuic Acid Mitigates Alcohol-Induced Decline of Alkaline Phosphatase

**FIGURE 5:**
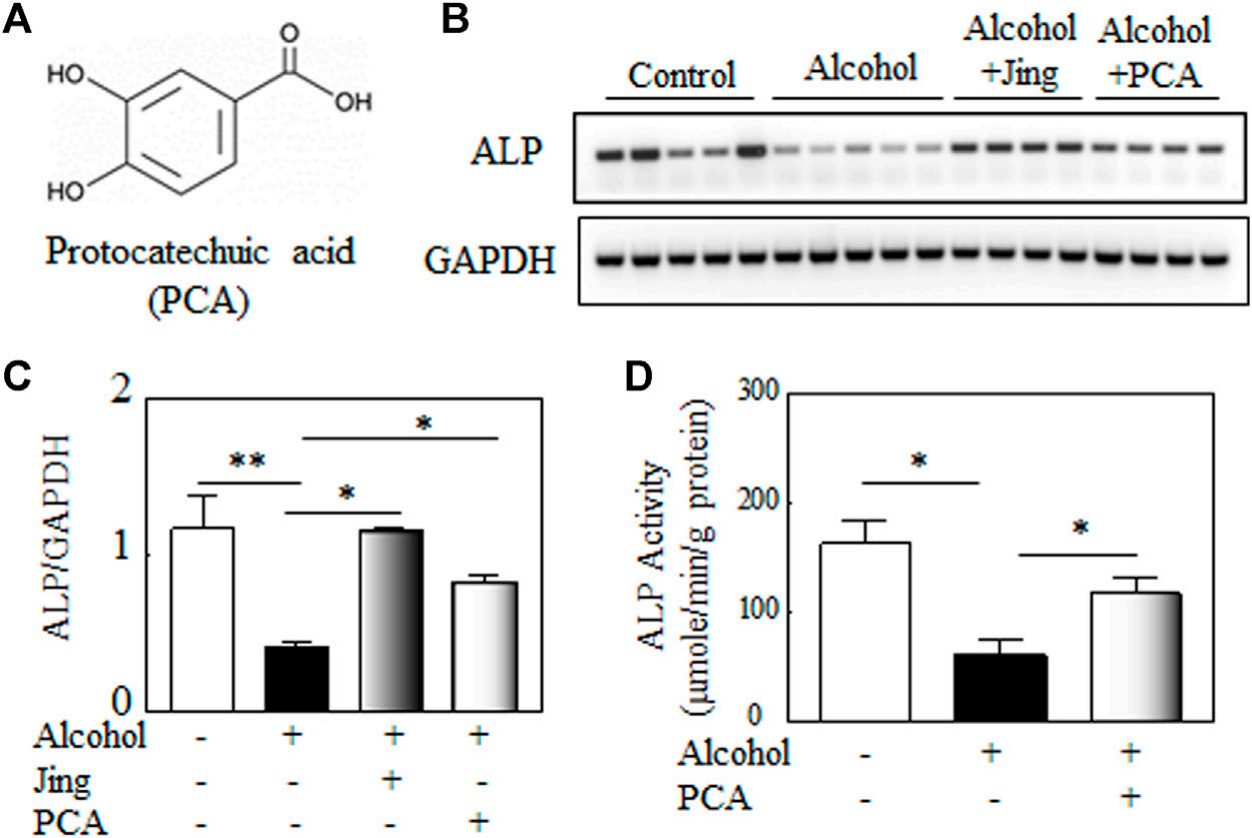
Protocatechuic acid (PCA) mitigates alcohol-induced decline of alkaline phosphatase (ALP). **(A)** The chemical structure of Protocatechuic acid. **(B)** Representative gel electrophoretogram of RT-PCR products shows *ALP* and *GAPDH*, the internal control, gene expression in the bone marrows of Balb/c male mice. **(C)** The quantitative analysis of *ALP* gene expression shows that Jing extracts and PCA mitigate the alcohol-induced decline of ALP gene expression *in vivo* (alcohol, *n* = 5, vs. control, *n* = 5, **p < 0.01; alcohol + Jing extracts, *n* = 4, or alcohol + PCA, *n* = 4 vs. alcohol, *p < 0.05; Mann-Whitney test). **(D)** The effects of PCA on ALP activity in murine pre-osteoblastic cell line MC3T3-E1 cells. PCA (100 μM) significantly alleviates the inhibitory effects of alcohol (500 mM) on ALP activity *in vitro* (osteogenic control or alcohol + PCA vs. alcohol, *p < 0.05; *n* = 4, Mann-Whitney test).

Among the 189 compounds identified by HPLC, LC/MS, and NMR (Cai et al., 2020) and 43 bioactive components identified by LC/MS (Hu et al., 2021) in Jing extracts, after a comprehensive literature review, we identify that Acteoside, Daidzin, Echinacoside, Eugenol, Ferulic acid, Hyperoside, Icariin, Oleanolic acid, and Protocatechuic acid have therapeutic potential for either bone or alcohol-related diseases (Table 2). To study the roles of bioactive compounds in the studied herbs in bone diseases, we selected Protocatechuic acid (Figure 5A) as a representative bioactive compound in the Jing extracts to test its effects on alkaline phosphatase and other bone metabolic factors in the bone marrow of Balb/c male mice and *in vitro* alkaline phosphatase activity in murine pre-osteoblastic cell line MC3T3-E1 cells.

Balb/c male mice (2-month-old) were orally administered 3.2 g/kg alcohol by gavage with or without 0.25 g/kg of Jing extracts or 50 mg/kg of PCA for 10 days, a time pint that alcohol damaged other organs, e.g., liver (Bertola et al., 2013), but not bones. Our data (Figures 5B,C) showed that as the Jing extracts, PCA significantly mitigates alcohol-induced decline of alkaline phosphatase gene expression in the bone marrow of Balb/c male mice. Jing extracts and PCA reduced the alcohol-induced elevated inflammatory factors, e.g., TNF-α and IL-1β in the bone marrow of Balb/c male mice (Supplementary Figure S6). To study the effects of PCA on alkaline phosphatase (ALP) activity, murine pre-osteoblastic cell line MC3T3-E1 cells were treated with or without alcohol and/or PCA in an osteogenic medium. Our data (Figure 5D) showed that PCA significantly alleviates the inhibitory effects of alcohol on ALP activity in MC3T3-E1 cells.

## Discussion

Alcohol is a nonessential diet component, and the overall impact of drinking on bone health, especially at moderate levels, is not well understood (Gaddini et al., 2016). Light to moderate alcohol consumption refers to two drinks or less in a day for men or one drink or less in a day for women per Dietary Guidelines for Americans (USDA and HHS, 2020). Epidemiological studies showed that light to moderate alcohol consumption is generally reported to be beneficial, resulting in higher bone mineral density (BMD) and reduced age-related bone loss, especially in men and postmenopausal women (Felson et al., 1995; Tucker et al., 2009; Sommer et al., 2013). However, heavy alcohol consumption is generally associated with decreased BMD, impaired bone quality, and increased fracture risk (Turner, 2000; Maurel et al., 2012; Mikosch, 2014; Gaddini et al., 2016). Similar to the epidemiological data, our preclinical studies showed that the effects of alcohol on bone health in mice are dose-and time-dependent. Our data showed that oral alcohol administration of a low dose of alcohol (0.8 g/kg body weight) significantly increased BMD in adult male mice (Figure 2); a low dose of oral alcohol administration by gavage (1.6 g/kg for 50 days) or higher doses (3.2 g/kg) for a shorter period of gavage time (30 days) have a neutral effect on bone mineral density in young adult Balb/c male mice (Figure 2). However, a high dose of alcohol administration (3.2 g/kg) with a longer gavage time (50 days) induces significant bone loss in young adult Balb/c male mice (Figure 2 and Figure 3); thus, we used 3.2 g/kg alcohol dose and the duration of 50 days to study the effects of TCM herbal extracts on alcohol-induced osteopenia.

Osteoporosis is the most common bone disease, representing a significant public health problem worldwide (Cosman et al., 2014; Wright et al., 2014; Zhou and Glowacki, 2017; Langdahl and Andersen, 2018; Zhou and Glowacki, 2018; Compston et al., 2019). There has been considerable progress in understanding postmenopausal (Type I) and senile (Type II) osteoporosis, the primary osteoporosis; however, gaps of knowledge still exist in understanding osteoporosis/osteopenia caused by alcoholism, one type of secondary osteoporosis. Alcohol-induced osteopenia/osteoporosis is distinct from primary osteoporosis; particularly, alcohol-induced osteopenia results mainly from decreased bone formation rather than increased bone resorption (Chakkalakal, 2005), which is confirmed in old male mice by our biomarker analysis (Figure 4C). Our biochemical study (Figure 4) showed that alcohol significantly reduced serum levels of bone formation biomarker osteocalcin but not the serum levels of bone resorption biomarker CTXI in the middle-aged/old Balb/c male mice, suggesting that chronic alcohol consumption inhibits bone formation but has no effects on bone resorption. There is a paucity of studies regarding alcohol-induced osteoporosis therapy (Abukhadir et al., 2013). Although the study found that abstinence can stop bone loss and the lost bone can be partially restored when alcohol abuse ends (Alvisa-Negrín et al., 2009), research indicates that the effects of heavy alcohol use on bone cannot be reversed, even if alcohol consumption is terminated (Sampson, 2002). Additional studies should examine whether alcohol-induced osteoporosis is reversible. Nevertheless, severe osteoporosis, especially alcohol-induced osteoporosis in the elders, needs osteoporotic management.

Due to the nature of alcohol-induced osteopenia resulted mainly from decreased bone formation rather than increased bone resorption (Chakkalakal, 2005 and Figure 4C), anabolic treatments for osteoporosis, such as Teriparatide (parathyroid hormone 1–34), Abaloparatide (a parathyroid hormone-related protein analog drug), and Romosozumab (a humanized antibody against sclerostin), may help address these unmet needs. Other anabolic anti-osteoporotic agents, which stimulate bone formation, such as vitamin D and estrogen, should prevent alcohol-induced bone loss and benefit bone loss recovery during alcoholism and abstinence. Our data of oral administration of Vitamin D in Balb/c male mice (Supplementary Figure S5) confirmed a preclinical study that Vitamin D supplementation prevents chronic liquid diet alcohol-induced bone loss in female C57BL/6J mice (Mercer et al., 2012), suggesting that our data of oral alcohol administration by gavage are comparable with the data obtained by Lieber-DeCarli liquid diet feeding.

Traditional Chinese medicine (TCM) formulas have a long history of use in the prevention and treatment of osteoporosis, and phytochemicals from TCM formulas offer great potential for the development of novel antiosteoporotic drugs (Zhang et al., 2016; Lin et al., 2017). One of the molecular mechanisms of TCM antiosteoporotic drugs is the potential of promoting osteoblast-mediated bone formation (An et al., 2016), making TCM a good candidate for alcohol-induced osteoporosis therapy. The Xian-Ling-Gu-Bao capsule (XLGB) is an effective traditional Chinese medicine prescription that is used for the prevention and treatment of osteoporosis in China (Wu et al., 2017; Tang et al., 2020; Ding et al., 2021). We used XLGB as the TCM antiosteoporotic medicine control in our experiments and showed that TCM XLGB might prevent alcohol-induced osteoporosis in young adult and middle-aged/old male mice (Supplementary Figure S5). Traditional Chinese medicines for the treatment of osteoporosis and osteoporotic fractures follow the TCM rule of “Kidneys Govern Bones,” which uses herbs that medicate bone by improving the functions of the kidney (An et al., 2016; Wang et al., 2016). The Bone-kidney axis is critical for bone health. Kidney dysfunction, e.g., Chronic kidney disease (CKD), results in mineral and bone disorder (CKD-MBD), a systemic condition that links disorders of mineral and bone metabolism to either one or all of the following: abnormalities of calcium, phosphorus, PTH, or vitamin D metabolism, abnormalities in bone turnover, mineralization, volume, linear growth or strength, and extraskeletal calcification (Zhou et al., 2013; Zhou and Glowacki, 2017). In this study, the traditional Chinese medicine herbal extracts or Jing extracts (Figure 1) are a combinative herbal prescription of nine Chinese herbal medicines, including *Astragalus*, *Cistanche deserticola*, *Dioscorea polystachya* (Chinese yam), *Lycium barbarum*, *Epimedium*, *Cinnamomum cassia*, *Syzygium aromaticum*, *Angelica sinensis*, and *Curculigo orchioides*. This TCM formula is used in a famous Chinese Herbal Liqueur, Chinese Jing Liqueur (Liu et al., 2011; Lu et al., 2017; Lu et al., 2018; Shan et al., 2018; Cai et al., 2020; Hu et al., 2021). According to TCM theory of “kidney is the origin of the congenital constitution”, this empirical formula of the nine TCM herbs was formulated for the purpose of “tonifying kidney.” Among the nine TCM herbs, *Cistanche deserticola* and *Epimedium* are used as the herbs to reinforce the kidney-yang, which supplemented by *Dioscorea polystachya* (Chinese yam) and *Lycium barbarum*, etc., to replenish kidney-yin (Liu et al., 2011; Lu et al., 2017; Lu et al., 2018; Shan et al., 2018). Preclinical and clinical studies demonstrated that studied herbal formulation (Jing extracts) has the properties of improving kidney-yang deficiency in rats and relieving main symptoms of patients with Kidney-Yang Deficiency Syndrome, anti-fatigue, and enhancing immunity in humans and animals (Liu et al., 2011; Lu et al., 2017; Lu et al., 2018; Shan et al., 2018; Cai et al., 2020; Hu et al., 2021). The assertion of “kidney governing bones” in “Huangdi Neijing—Yellow Emperor Canon of Internal Medicine” has been confirmed by an increasing body of scientific data (Ju et al., 2014; Wang et al., 2016). Our data showed that the Chinese herbal medicine extracts prevent alcohol-induced osteopenia, as demonstrated by bone morphological and biochemical analysis, in both young adult and middle-aged male mice (Figures 3, 4), which may due to its kidney-tonifying property *via* kidney governing bones. The preventing mechanism of the phytomedicines in Jing extracts on alcohol-induced osteoporosis needs additional study.

The TCM herbs used in this study and the TCM extracts’ bioactive compounds have therapeutic potentials for bone diseases (Tables 1, 2). *Astragalus membranaceus* (Kang et al., 2013), *Astragalus membranaceus* and *Cinnamomum cassia* plus *Phellodendron amurense* (Huh et al., 2015), *Epimedium brevicornum* (Zhang et al., 2006) or a combination of *Astragalus membranaceus*, *Angelica sinensis*, and *Epimedium brevicornum* (Xie et al., 2012), *Syzygium aromaticum* (Karmakar et al., 2012) and *Angelica sinensis* (Lim and Kim, 2014) reverse bone loss in a rat model of postmenopausal osteoporosis. *Cistanche deserticola* has antiosteoporotic activity in type I osteoporotic rats (Zhang et al., 2021) and type II osteoporotic mice (Wang et al., 2021), which may result in the stimulation of bone formation (Li et al., 2012). *Dioscorea spongiosa* prevents glucocorticoid-induced osteoporosis in rats (Han et al., 2016) and aging-induced osteoporosis (Tikhonova et al., 2015). *Lycium barbarum* ameliorates osteoporosis in OVX mice (Kim et al., 2017). The analyses of chemical components of the Chinese herbal extracts we used in this study by LC/MS and NMR (Hu et al., 2021; Cai et al., 2020) showed that herbal extracts contain a variety of saponins, flavonoids, active polysaccharides, and other functional factors including amino acids, organic acids, trace elements and other nutrients that are required by the human body. Among the 189 compounds detected by LC/MS and NMR (Cai et al., 2020) and 43 bioactive components detected by LC/MS (Hu et al., 2021) from the Chinese herbal extracts used in this study, Acteoside, Calycosin, Daidzin, Echinacoside, Epimedin, Eugenol, Ferulic acid, Hyperoside, Icariside, Icariin, Oleanolic acid, Protocatechuic acid, Sagittatoside, and Ligustilide, etc. are antiosteoporotic compounds and some of these compounds have the therapeutic potential for alcohol use disorder (Table 2; the chemical structures of these bioactive compounds in Supplementary Figure S7). For example, icariin represents a class of flavonoids with bone-promoting activity, which could be used as a potential treatment for postmenopausal osteoporosis (Wang et al., 2018). These antiosteoporotic compounds may prevent alcohol-induced osteopenia by acting alone or in a synergistic manner. Nuclear factor erythroid-derived 2-related factor-2 (Nrf2) is a master regulator of oxidative stress and a critical antiosteoporotic factor (Chen et al., 2021). Cai et al. (2020) reported that Chinese herbal extracts activate Nrf2, implying that Nrf2 may be involved in the preventive effects of Chinese herbal extracts (Jing extracts) on alcohol-induced osteoporosis.

The bone marrow is an alcohol sensitive organ (Wahl et al., 2007); hematopoietic cells and bone cells could be extrinsic factors for each other in the bone marrow niche (Zhou, 2015), which makes bone marrow an ideal organ to study the bone metabolic factors, such as ALP and TNF-α, etc. (Wahl et al., 2007; Zhou, 2015). Our data shows that Protocatechuic acid, a natural phenolic acid in Jing extracts, mitigates *in vivo* alcohol-induced decline of Alkaline phosphatase (ALP) gene expression (Figure 5) and elevated inflammatory factors (Supplementary Figure S6) in the bone marrow of male Balb/c mice. The murine pre-osteoblastic cell line MC3T3-E1 is a widely used in osteoblast biology (Hwang and Horton, 2019). In this study, MC3T3-E1 cells were treated with or without alcohol and/or PCA to study the effects of PCA on alcohol-induced inhibitory on ALP activity *in vitro*. Our data (Figure 5D) showed that PCA significantly alleviates the inhibitory effects of alcohol on ALP activity in MC3T3-E1 cells. Protocatechuic acid (PCA) is antioxidant and anti-inflammatory (Kakkar and Bais, 2014). Oxidative stress and inflammation are two common mechanisms involved in alcohol toxicity. PCA may prevent alcohol-induced bone injury via antioxidant and anti-inflammatory pathways. In this study, we used PCA as a representative bioactive compound in the Jing extracts. A typical TCM or any ethnomedicine formula has multiple ingredients that synergize in treatment for the symptoms of a disease, which have greater efficacy and safety than a single drug in clinical practices, possibly due to the multiple ingredients synergistic interactions and mutual detoxification. The other bioactive compounds, e.g., Acteoside, Daidzin, Echinacoside, Eugenol, Ferulic acid, Hyperoside, Icariin, and Oleanolic acid, etc. (Table 2), and their synergizing in treatment for alcohol-induced bone loss need additional investigations.

The TCM herbs used in this study also have therapeutic potentials for alcohol use disorder, especially alcoholic liver damage (Table 1). The chemical composition of TCM herbal extracts includes Acteoside, Daidzin, Echinacoside, Eugenol, Ferulic acid, Hyperoside, Icariin, Oleanolic acid, and Protocatechuic acid have therapeutic potential for alcohol-related diseases (Table 2). Osteoporosis is a common complication of many types of liver disease (Nakchbandi and van der Merwe, 2009). Alcohol consumption is an independent risk factor for the onset of osteoporosis, which is prevalent in patients with alcoholic liver disease (López-Larramona et al., 2013). The roles of TCM herbal extracts and their bioactive compounds in the alcohol-induced dysfunction of the liver-bone axis need additional study.

## Conclusion

In summary, our study in pharmacognosy suggests that traditional Chinese medicine herbal extracts prevent chronic excessive alcohol consumption-induced osteopenia in young adult and middle-aged/old male mice, which indicates that TCM might prevent alcohol-induced osteoporosis. Based on our data and previous reports (Liu et al., 2011; Lu et al., 2017; Lu et al., 2018; Cai et al., 2020; Hu et al., 2021), the potential protective mechanism of traditional Chinese medicine herbal extracts in alcohol-induced osteoporosis may involve the kidney-bone axis to prevent alcohol-induced bone loss and the direct antiosteoporotic potential of the bioactive compounds in the Chinese herbal medicines. Protocatechuic acid, a natural phenolic acid in Jing extracts, mitigates *in vivo* and *in vitro* alcohol-induced decline of alkaline phosphatase gene expression and activity. Additional analysis of the bioactive compounds in Chinese herbal medicines may identify antiosteoporotic compounds as drug candidates for preventing or treating alcohol-induced osteopenia/osteoporosis.

## Data Availability

The original contributions presented in the study are included in the article/Supplementary Material, further inquiries can be directed to the corresponding authors.

## Abbreviations

ALP, Alkaline phosphatase; CTXI, Cross-Linked C-Telopeptide of Type I Collagen; C. BMD, cortical bone mineral density; ELISA, enzyme-linked immunosorbent assay; HPLC, High-performance liquid chromatography; LC/MS, liquid chromatography-mass spectrometry; μ-CT, micro-computed tomography; NMR, nuclear magnetic resonance; OC, osteocalcin; Tb. BMD, trabecular bone mineral density; Tb. BV/TV, tibia trabecular relative bone volume; Tb. Th, trabecular thickness; Tb. Sp, trabecular space; Tb. N, trabecular number; TCM, traditional Chinese medicine.
