# Supplementary material for: A Traditional Chinese Medicine Plant Extract Prevents Alcohol-Induced Osteopenia
Source: Front Pharmacol. 2021 Dec 15;12:754088. doi: 10.3389/fphar.2021.754088 (PMC8730326; doi:10.3389/fphar.2021.754088)
Supplement: Supplementary file 1 [file Presentation1.pdf]

Supplemental Materials | Front. Pharmacol. 12:754088.

doi: 10.3389/fphar.2021.754088

A Traditional Chinese medicine plant extract prevents alcohol-induced osteopenia

Dongyang Qian, Hui Zhou, Pan Fan, Tao Yu, Anish Patel, Morgan O'Brien, Zhe Wang, Shiguang Lu, Guoqiang Tong, Yimin Shan, Lei Wang, Yuan Gao, Yuan Xiong, Lily Zhang, Xin Wang, Yuancai Liu*, Shuanhu Zhou*

* Correspondence: Dr. Shuanhu Zhou, (szhou@bwh.harvard.edu; shuanhuzhou@gmail.com), Director of Skeletal Biology Laboratory, Department of Orthopedic Surgery, Brigham and Women's Hospital, Harvard Medical School, Boston, Massachusetts, USA; Dr. Yuancai Liu. (lyc@jingpai.com), Jing Brand Research Institute, Jing Brand Co., Ltd., Daye, Hubei, China.

Supplemental Materials

Traditional Chinese Medicine Herbal Extracts (Jing Extracts) Administration in Mice

It was recommended that the adult human dose of the traditional Chinese medicine herbal extracts (Jing extracts) in 35% v/v liquor is 100 mL per day (Liu et al., 2008; Liu et al., 2011; Lu et al., 2017, 2018; Shan et al., 2018). The mouse dose is equivalent to the human dose by multiplying the human-mouse dose conversion factor of 12.3 (Wojcikowski and Gobe, 2014; Nair and Jacob S., 2016), 100 mL per day for a 60 kg adult human (1.67 mL/kg) nearly equals to 20 ml/kg of mouse dose (0.4 ml of a 20 g mouse). Taking into account that the commonly used mouse gavage volume is 10 mL/kg (0.2 mL for a 20g mouse), we defined the first mouse dose of Chinese herbal extracts (0.2 mL for a 20 g mouse), which is equivalent to a human dose of 100 mL of 6.28 g/L of Chinese herbal extracts for a 60 kg adult human, as the low dose (0.125 g/kg body weight for a 20 g mouse with 0.2 mL oral administration by gavage of 12.5 g/L of Chinese herbal extracts in 40% v/v alcohol); the second and third doses contained 0.25 g/kg (25 g/L of Chinese herbal extracts in 40% v/v alcohol) and 0.50 g/kg (50 g/L of Chinese herbal extracts in 40% v/v alcohol) of Chinese herbal extracts respectively. We tested the effects of these three doses of Chinese herbal extracts on alcohol-induced bone loss in Balb/c male mice. After 50 days of orally administering 3.2 g/kg of alcohol with/without Chinese herbal extracts by gavage, the mice were sacrificed for bone morphological and biochemical analysis.

doi: 10.3389/fphar.2021.754088

Supplemental Figure S1

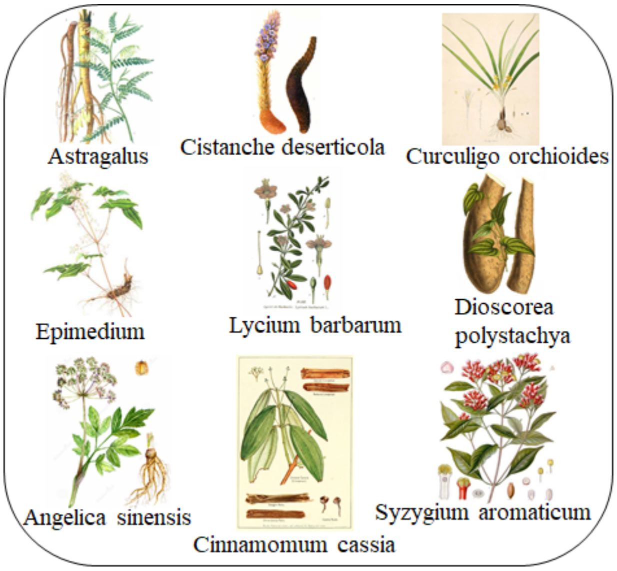

Suppl. Fig. S1 The references of the photos of TCM herbs in Fig. 1.
Astragalus membranaceus (https://emeryherbals.com/herb-of-the-month-astragalus/),
Cinnamomum cassia (https://commons.wikimedia.org/wiki/File:Cinnamon-cassia.png),
Cistanche deserticola (http://www.nutragreenbio.com/product/cistanche-extract),
Lycium barbarum (https://pfaf.org/user/plant.aspx?latinname=Lycium+barbarum),
Epimedium brevicornum (http://www.pzmybio.com/content-45-150-1.html),
Angelica sinensis (https://www.goldenpoppyherbs.com/dong-quai-materia-medica/),
Dioscorea polystachya (http://www.itmonline.org/arts/dioscorea.htm),
Curculigo orchioides (http://plantillustrations.org/illustration.php?id_illustration=61224)
Syzygium aromaticum (https://en.wikipedia.org/wiki/Clove).

Supplemental Figure S2

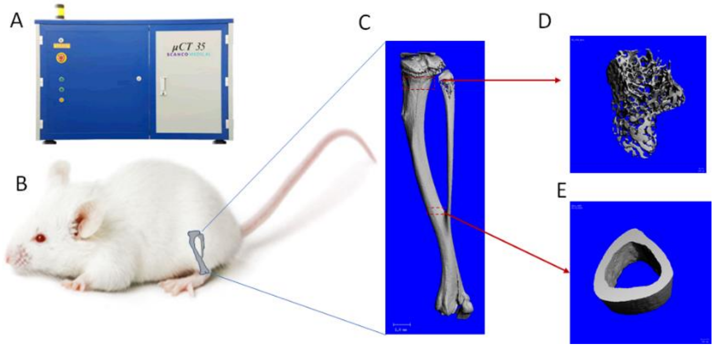

Suppl. Fig. S2 μ-CT analysis of tibia. (A) μ-CT 35 (Scanco Medical, Switzerland). (B) Tibia of Balb/c mice was used for bone morphological analysis with μ-CT (the 3-D image of the tibia was modified from

http://skeletal.group.shef.ac.uk/www/wp-content/uploads/2015/07/tibia-scanco.jpg). (C) Regions of interest for the analysis of trabecular and cortical bone. (D) The scanned region proximal to the growth plate and extending 1.4 mm was selected for trabecular bone analysis (indicated with a box in C). (E) A second region 0.6 mm in length and centered at the midpoint of the tibia was used to calculate diaphyseal parameters (indicated with a box in C).

Supplemental Figure S3

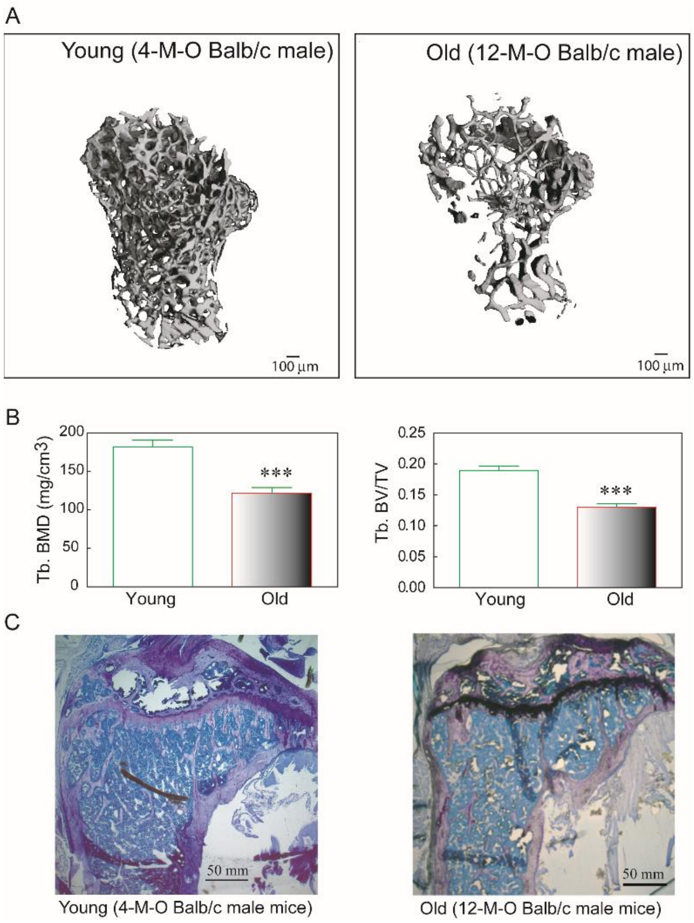

Suppl. Fig. S3 The different bone structures between young adult and middle-aged/old Balb/c male mice. (A) The representative μ-CT 3-D microstructures of trabecular bone of young adult (4-month-old) and middle-aged/old (12-month-old) Balb/c male mice; bars represent 100 μm; 3-D microstructural properties of the tibia were calculated using software supplied by the manufacturer. (B) Trabecular bone mineral density (Tb. BMD) in old mice (n=9) was significantly lower than in the young adult mice (n=18) (***p<0.001, t-test); the quantitative analysis of tibia trabecular relative bone volume (Tb. BV/TV) (***p<0.001, t-test). (C) The representative toluidine blue staining of the tibia of young and old Balb/c male mice, showing that the tibia trabecular number in old mice is less than the young adult mice.

doi: 10.3389/fphar.2021.754088

Supplemental Figure S4

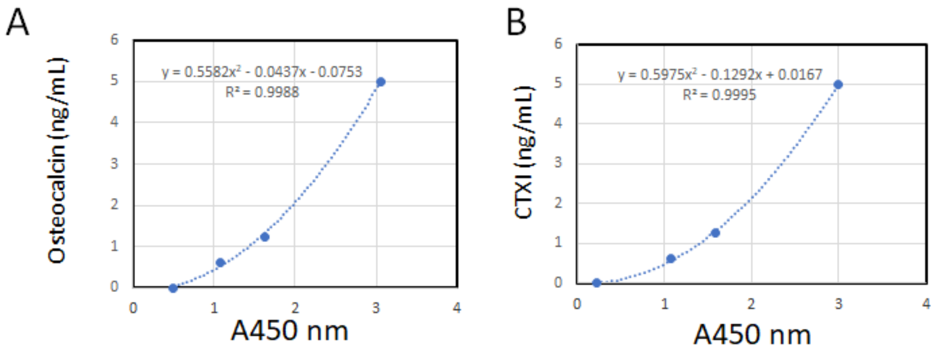

Suppl. Fig. S4 ELISA standard curves. (A) The standard curve of the mouse osteocalcin ELISA kit (MBS2701836, MyBioSource, San Diego, CA); the standard curve concentrations used for this ELISA were 0 ng/mL, 0.625 ng/mL, 1.25 ng/mL, 5 ng/mL of osteocalcin. (B) The standard curve of mouse Cross-Linked C-Telopeptide Of Type I Collagen (CTXI) ELISA Kit (MBS453660, MyBioSource, San Diego, CA); the standard curve concentrations used for this ELISA were 0 ng/mL, 0.625 ng/mL, 1.25 ng/mL, 5 ng/mL of CTXI from the ELISA kit.

Supplemental Figure S5

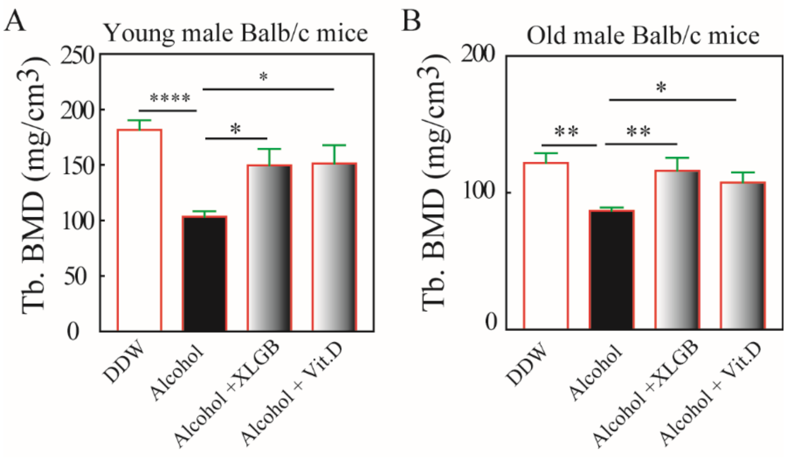

Suppl. Fig. S5 The effects of XLGB and vitamin D on alcohol-induced osteopenia. We used Xianling Gubao (XLGB), a TCM for osteoporosis treatment, and vitamin D as positive controls in our experiments. (A) The young adult Balb/c male mice (2-month-old) were orally administrated 3.2 g/kg body weight of alcohol with or without 0.4 g/kg of Xianling Gubao (XLGB, Sinopharm Group Tongjitang Pharmaceuticals Co Ltd, Guizhou, China), a TCM for osteoporosis patients (Wu et al., 2017); XLGB contains Epimedium brevicornum, Dipsacus fullonum, Salvia miltiorrhiza, Anemarrhena asphodeloides, Psoralea corylifolia, and Rehmannia glutinosa (Ding et al., 2021) and the 0.4 g/kg mouse dose equals to the recommended human dose (twice per day, 3 capsules of 0.3 g per capsule), or 2000 IU/kg of vitamin D_3, which is known to prevent the alcohol-induced osteopenia (Mercer et al., 2012). After 50 days, XLGB and Vitamin D3 significantly prevented alcohol-induced osteopenia in young adult male mice (about 4-

doi: 10.3389/fphar.2021.754088

month-old) (*p<0.05, Alcohol+ XLGB, n=6 vs. Alcohol, n=8; *p<0.05, Alcohol + Vit. D, n=5, vs. Alcohol, n=8; ANOVA). (B) The middle-aged/old Balb/c male mice (10-month-old) were orally administrated 3.2 g/kg body weight of alcohol with or without 0.4 g/kg of XLGB or 2000 IU/kg of vitamin D_3; after 50-day treatment, both XLGB and Vitamin D3 significantly prevented alcohol-induced osteopenia in old male mice (**p<0.01, Alcohol + XLGB, n=5, vs. Alcohol, n=8; *p<0.05, Vit. D + Alcohol, n=7, vs. Alcohol, n=8; ANOVA).

Supplemental Figure S6

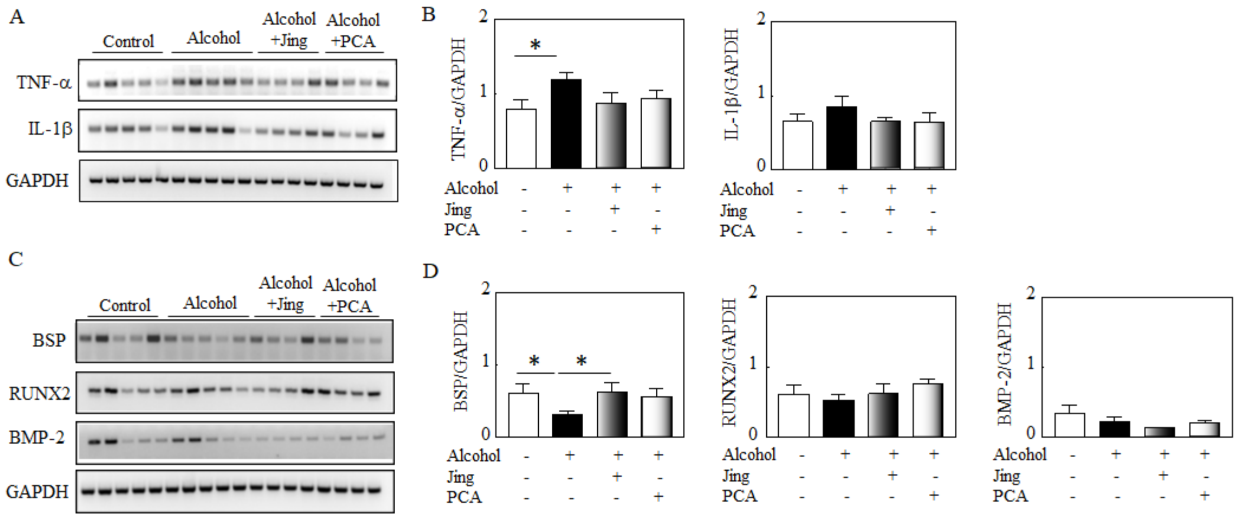

Suppl. Fig. S6 Protocatechuic acid (PCA) mitigates alcohol-induced changes in pro-inflammatory cytokines and osteogenic factors. (A) Representative gel electrophoretogram of RT-PCR products shows pro-inflammatory cytokines *TNF-α* and *IL-1β* gene expression in the bone marrows of Balb/c male mice. *GAPDH* was used as the internal control. (C) The quantitative analysis of *TNF-α* and *IL-1β* gene expression shows that Jing extracts and PCA have the treads to mitigate the alcohol-induced increase of *TNF-α* and *IL-1β* gene expression *in vivo* (alcohol, n=5, vs. control, n=5, *p<0.05; Mann-Whitney test). (C) Representative gel electrophoretogram of RT-PCR products shows osteogenic factors Bone Sialoprotein (*BSP), RUNX2* and *BMP-2* gene expression in the bone marrows of Balb/c male mice. *GAPDH* was used as the internal control. (D) The quantitative analysis of *BSP, RUNX2* and *BMP-2* gene expression shows that Jing extracts and PCA have the treads to mitigate the alcohol-induced decline of *BSP* (alcohol, n=5, vs. control, n=5, *p<0.05; alcohol + Jing extracts, n=4 vs. alcohol, *p<0.05; Mann-Whitney test); alcohol has no significant influence on *RUNX2* and *BMP-2* gene expression in the bone marrows of Balb/c male mice.

Supplemental Materials | Front. Pharmacol. 12:754088.

doi: 10.3389/fphar.2021.754088

Supplemental Figure S7

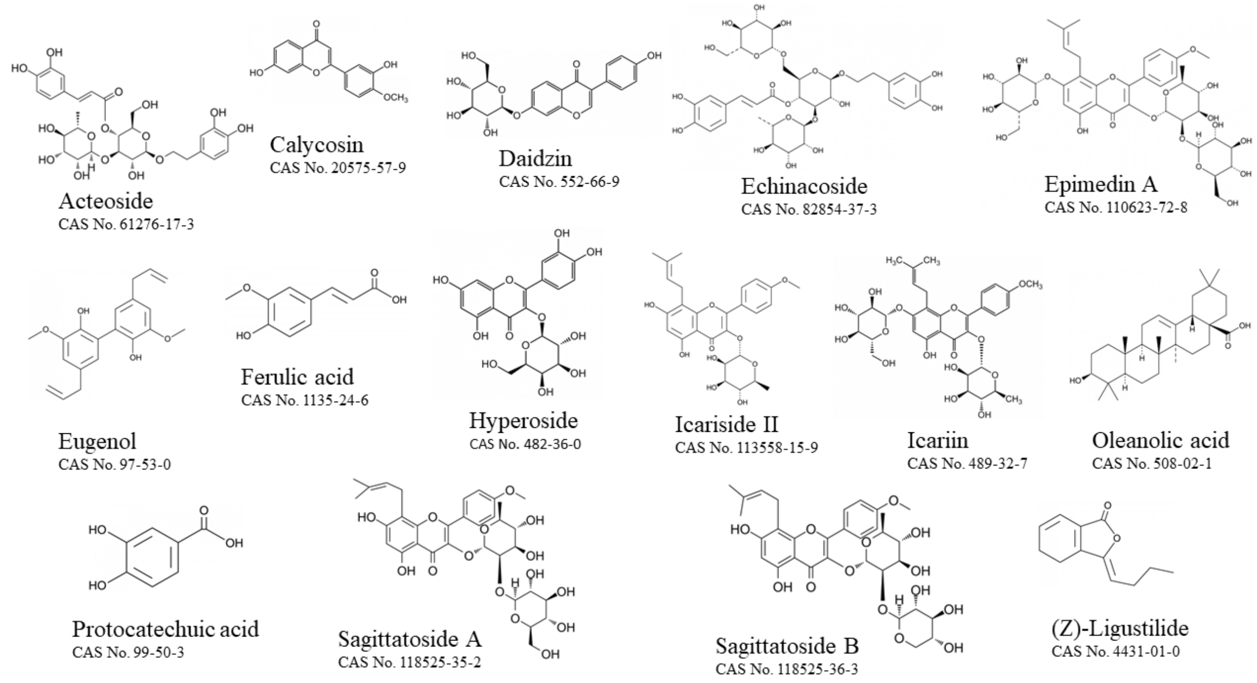

Suppl. Fig. S7 The chemical structures of herbal bioactive compounds. The chemical structures and Chemical Abstracts Service Registry Number (CAS No.) reference www.biomol.com, www.chemsrc.com/en, www.medchemexpress.com, www.chemfaces.com, and www.selleckchem.com.

Supplemental Materials | Front. Pharmacol. 12:754088.

doi: 10.3389/fphar.2021.754088

Suppl. TABLE S1 The traditional use of the studied herbs in Jing extracts

Herbs*	Traditional use of the studied herbs§
Astragalus mongholicus Bunge<br>(Astragalus)<br>Chinese: Huang Qi	To tonify qi and strengthen the superficial resistance, induce diuresis and promote drainage of pus and growth of new tissue.
Cistanche deserticola Y. C. Ma<br>(Cistanche deserticola)<br>Chinese: Rou Cong Rong	To reinforce kidney-yang, replenish essence and blood, and induce laxation.
Dioscorea polystachya Turcz.<br>(Dioscorea polystachya, Chinese yam)<br>Chinese: Shan Yao	To replenish the spleen and stomach, engender fluids and benefit the lung, and strengthen the kidney and restrain seminal discharge.
Lycium barbarum L.<br>(Lycium barbarum, Chinese wolfberry or Goji berry)<br>Chinese: Gou Qi	To enrich the liver and the kidney, replenish vital essence, and improve vision.
Epimedium brevicornu Maxim<br>(Epimedium, Herba Epimedii)<br>Chinese: Yin Yang Huo	To reinforce kidney-yang, strengthen the tendons and bones, and dispel wind and dampness.
Cinnamomum cassia (L.) J. Presl<br>(Cinnamomum cassia, Chinese Cinnamon),<br>Chinese: Rou Gui	To tonify fire and assist yang, and lead the fire back to the kidney, dispel cold and relieve pain, and activate blood circulation, and stimulate menstrual discharge.
Syzygium aromaticum Merr. and L.M.Perry<br>(Syzygium aromaticum, clove)<br>Chinese: Ding Xiang	To warm the middle-energizer, check the adverse rise of the stomach-qi, and restore the kidney-yang.
Angelica sinensis (Oliv.) Diels<br>(Angelica sinensis, Chinese angelica)<br>Chinese: Dang Gui	To tonify and activate blood, regulate menstruation, relieve pain, moisten the intestines and relax bowels.
Curculigo orchioides Gaertn.<br>(Curculigo orchioides, Rhizoma curculiginis, Curculigo Rhizome)<br>Chinese Pinyin: Xian Mao	To reinforce kidney-yang, strengthen the tendons and bones, and dispel cold-dampness.

* The Chinese medicinal plant names referred to The Plant List (http://www.theplantlist.org/), Medicinal Plant Names Services of Royal Botanic Gardens, Kew (https://mpns.science.kew.org/mpns-portal/), or NCBI taxonomy database (https://www.ncbi.nlm.nih.gov/taxonomy).
§ Chinese herbal medicine database, Hong Kong Polytechnic University (https://herbaltcm.sn.polyu.edu.hk/).

doi: 10.3389/fphar.2021.754088

Suppl. Table S2 PCR primers used in this study

Gene	Primers	Amplicon length (bp)	Reference
ALP	Forward: 5'-catgacatcccagaaagac-3'<br>Reverse: 5'-gttgtgagcgtaatctacc-3'	226	Zhou et al. 2001
TNF-α	Forward: 5'-tctcatcagttctatggccc-3'<br>Reverse: 5'-gggagtagacaaggtacaac-3'	212	Van Bezooijen et al., 1998
IL-1β	Forward: 5'-ttgacggaccccaaaagatg-3'<br>Reverse: 5'-agaaggtgctcatgtcctca-3'	204	Van Bezooijen et al., 1998
BSP	Forward: 5'-aaagtgaaggaaagcgacga-3'<br>Reverse: 5'-gttccttctgcacctgcttc-3'	214	Su et al., 2020
RUNX2	Forward: 5'-atccatccactccaccacgc-3'<br>Reverse: 5'-aagggtccactctggctttgg-3'	372	Hegert et al., 2002
BMP-2	Forward: 5'-catccagccgacccttg-3'<br>Reverse: 5'-ctctcccactgacttgtg-3'	505	Zhou et al., 2001
GAPDH	Forward: 5'-accacagtccatgccatcac-3'<br>Reverse: 5'-tccaccaccctgttgctgta-3'	452	Zhou et al., 2016

References

Hegert, C., Kramer, J., Hargus, G., Müller, J., Guan, K., Wobus, A.M., Müller, P.K., Rohwedel, J. (2002). Differentiation plasticity of chondrocytes derived from mouse embryonic stem cells. J Cell Sci. 115, 4617-4628. doi: 10.1242/jcs.00171.

Su, S., Ren, Y., Zhang, Y., Zhao, Y., Xiao, E. (2020). Antibiotics Disturb Dentin Formation and Differentiation of Dental Pulp Stem Cells: The Role of Microbiota in Cellular Turnover of Mouse Incisor. Stem Cells Int. 2020, 5154707. doi: 10.1155/2020/5154707.

Van Bezooijen, R.L., Farih-Sips, H.C., Papapoulos, S.E., Löwik, C.W. (1998). IL-1alpha, IL-1beta, IL-6, and TNF-alpha steady-state mRNA levels analyzed by reverse transcription-competitive PCR in bone marrow of gonadectomized mice. J Bone Miner Res. 13, 185-194. doi: 10.1359/jbmr.1998.13.2.185.

Zhou, S., Zilberman, Y., Wassermann, K., Bain, S.D., Sadovsky, Y., Gazit, D. (2001). Estrogen modulates estrogen receptor alpha and beta expression, osteogenic activity, and apoptosis in mesenchymal stem cells (MSCs) of osteoporotic mice. J Cell Biochem. Suppl 36, 144-155. doi: 10.1002/jcb.1096.

Zhou, S., Thornhill, T.S., Meng, F., Xie, L., Wright, J., Glowacki, J. (2016). Influence of osteoarthritis grade on molecular signature of human cartilage. J Orthop Res. 34, 454-462. doi: 10.1002/jor.23043.

doi: 10.3389/fphar.2021.754088

Supplemental Figure S8

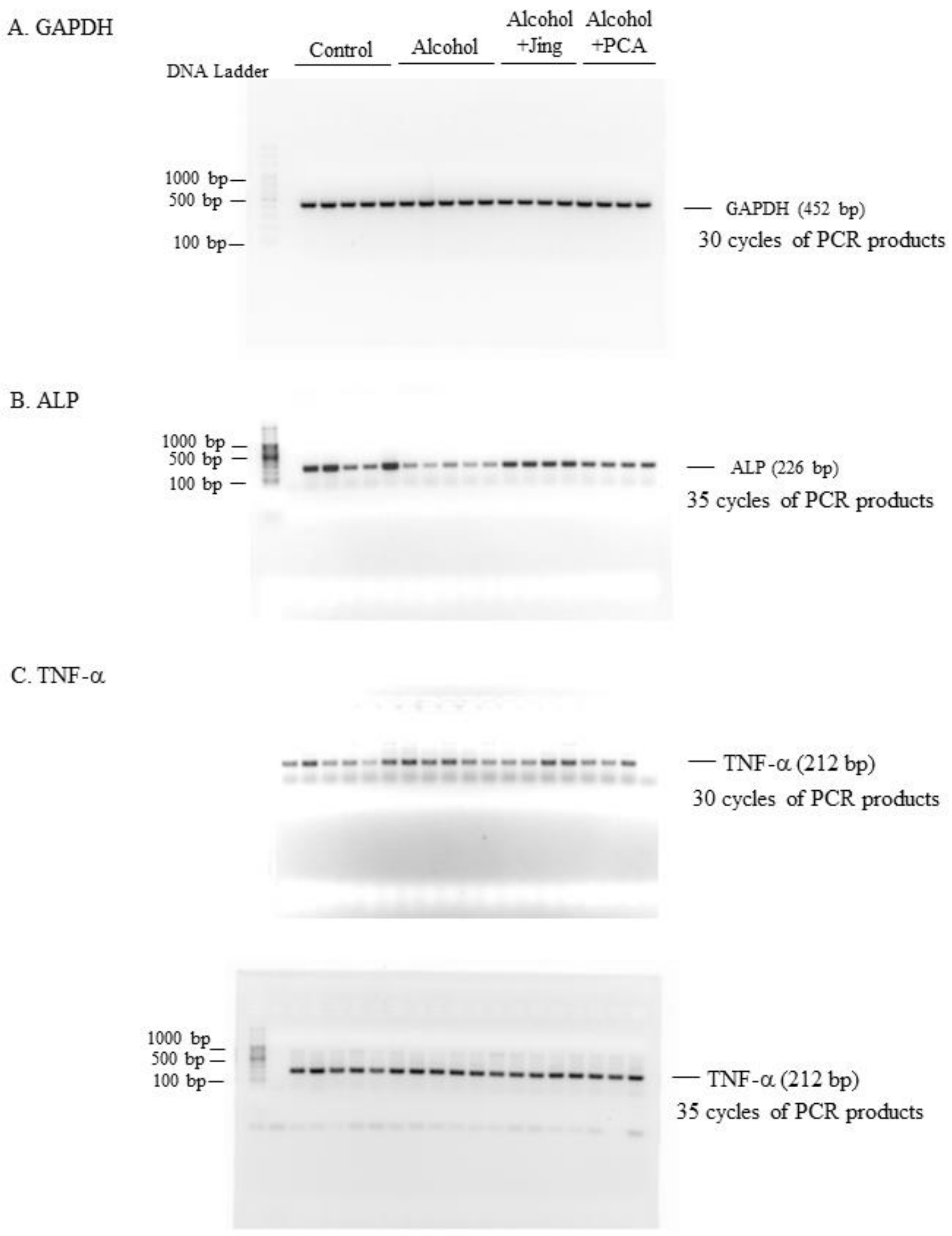

doi: 10.3389/fphar.2021.754088

D. IL-1β

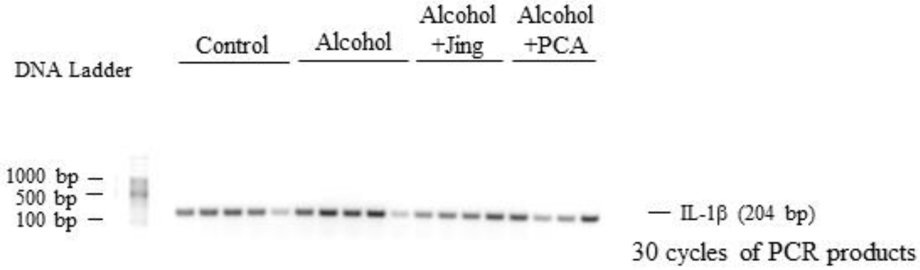

E. BSP

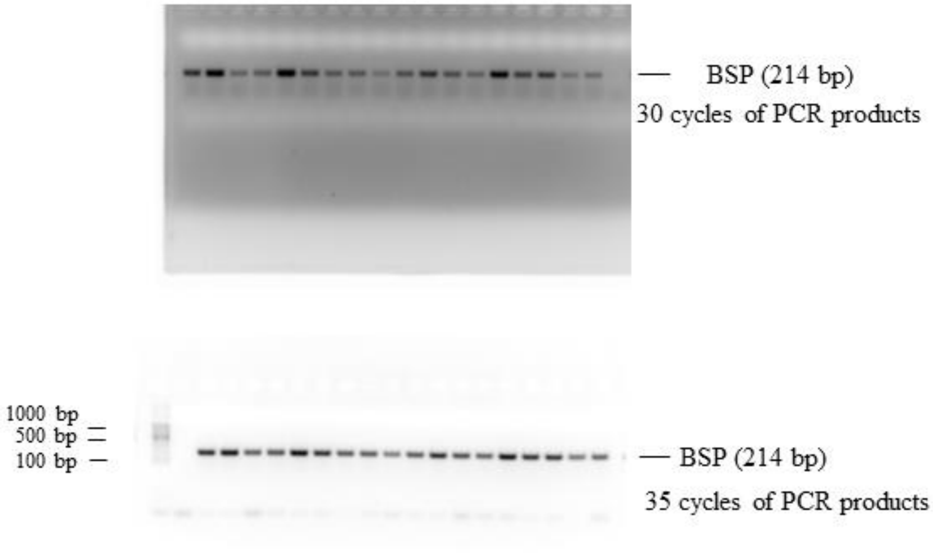

F. RUNX2

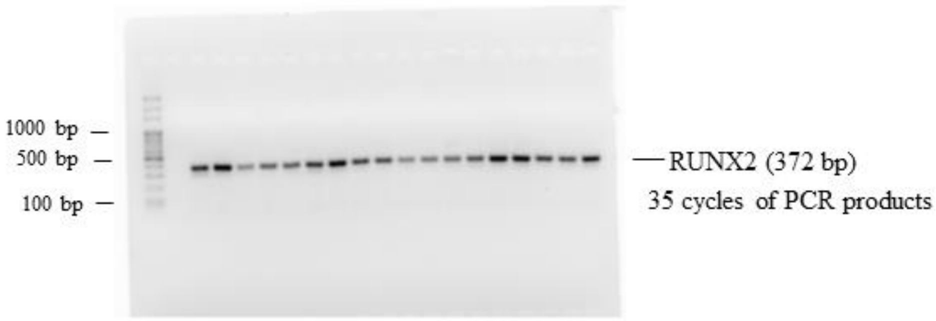

doi: 10.3389/fphar.2021.754088

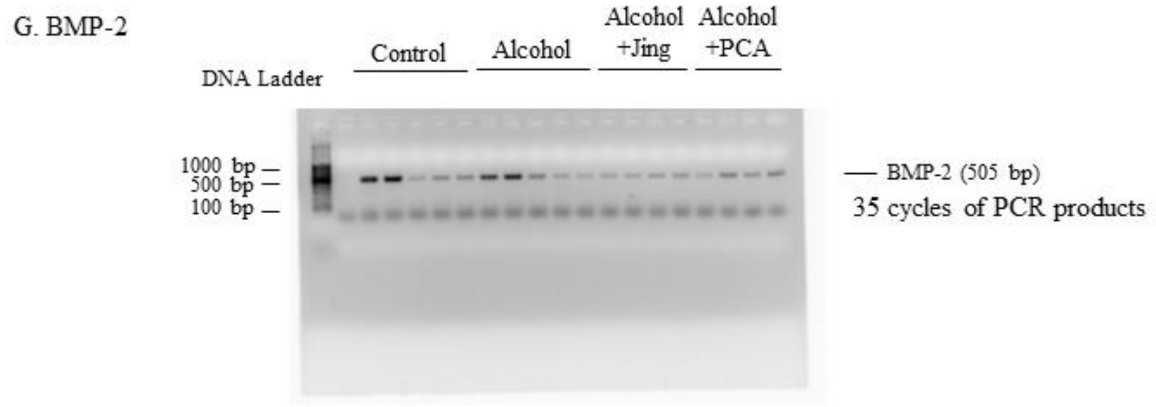

Suppl. Figure S8. The original PCR gel images (inverted images of ethidium bromide/agarose gels) of Fig. 5 and Suppl. Fig. S6. The DNA ladder marker was purchased from DNAland Scientific (Cat. No. GBR104, Baton Rouge, LA); 1000 bp, 500 bp, and 100 bp were indicated. The gene name and its PCR product size (bp) were displayed on the right side of each gel image.
